# Studies and Application of Sialylated Milk Components on Regulating Neonatal Gut Microbiota and Health

**DOI:** 10.3389/fnut.2021.766606

**Published:** 2021-11-10

**Authors:** Yushuang Wang, Xiaolei Ze, Binqi Rui, Xinke Li, Nina Zeng, Jieli Yuan, Wenzhe Li, Jingyu Yan, Ming Li

**Affiliations:** ^1^College of Basic Medical Science, Dalian Medical University, Dalian, China; ^2^Science and Technology Centre, By-Health Co., Ltd., Guangzhou, China; ^3^Key Laboratory of Separation Science for Analytical Chemistry, Dalian Institute of Chemical Physics, Chinese Academy of Sciences (CAS), Dalian, China

**Keywords:** breast milk, sialic acids, oligosaccharides, glycoconjugates, new-born, gut microbiota

## Abstract

Breast milk is rich in sialic acids (SA), which are commonly combined with milk oligosaccharides and glycoconjugates. As a functional nutrient component, SA-containing milk components have received increasing attention in recent years. Sialylated human milk oligosaccharides (HMOs) have been demonstrated to promote the growth and metabolism of beneficial gut microbiota in infants, bringing positive outcomes to intestinal health and immune function. They also exhibit antiviral and bacteriostatic activities in the intestinal mucosa of new-borns, thereby inhibiting the adhesion of pathogens to host cells. These properties play a pivotal role in regulating the intestinal microbial ecosystem and preventing the occurrence of neonatal inflammatory diseases. In addition, some recent studies also support the promoting effects of sialylated HMOs on neonatal bone and brain development. In addition to HMOs, sialylated glycoproteins and glycolipids are abundant in milk, and are also critical to neonatal health. This article reviews the current research progress in the regulation of sialylated milk oligosaccharides and glycoconjugates on neonatal gut microbiota and health.

## Introduction

**Figure 1:**
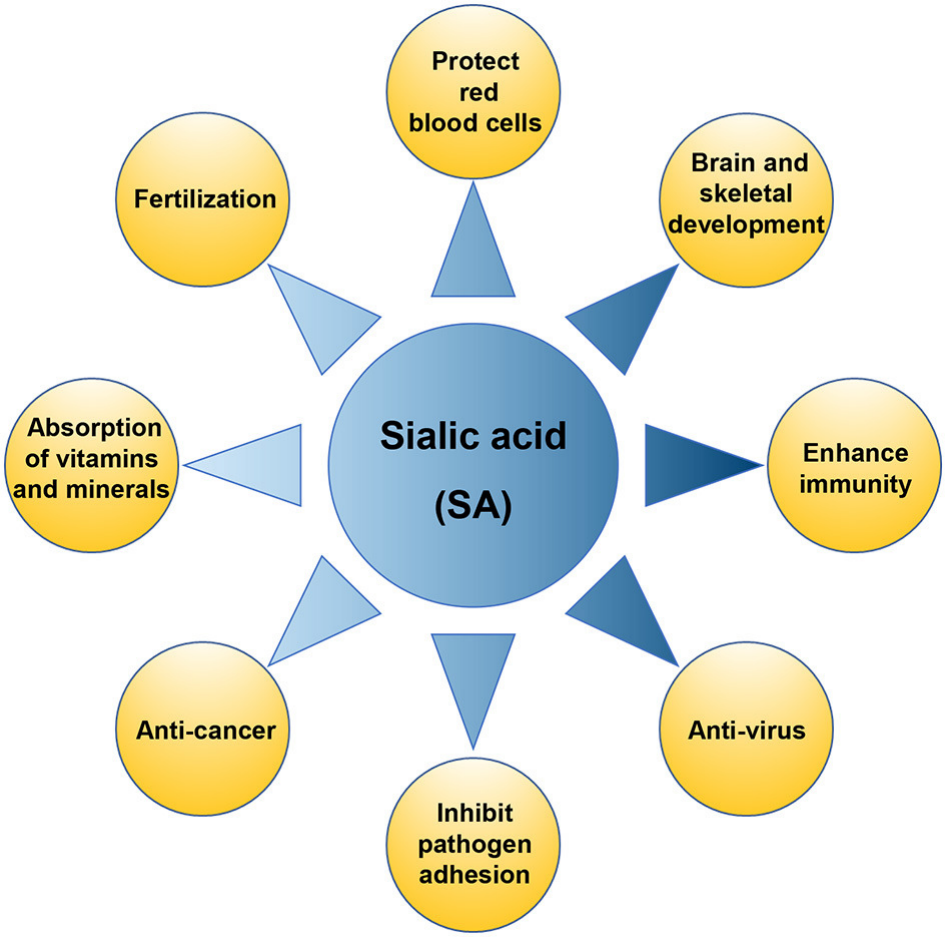
The physiological function of sialic acid (SA).

Sialic acid (SA), known as N-acetylneuraminic acid (Neu5Ac), was originally isolated from bovine mandibular salivary gland mucin by a scientist named Blix. It is a negatively charged acidic monosaccharide containing nine carbon atoms, with a free carboxyl group at the anomeric carbon C2, and an N-acetyl group at C5 (1–3). More than 50 forms of SA have been found in nature, of which more than 15 have been identified in humans (4). SA is an essential functional sugar with multiple known roles (Figure 1), which are crucial for infant health, promoting the development of the brain and nervous system, and enhancing immunity. SA can also increase the absorption of minerals and vitamins in the intestinal tract and promote bone development (5). SA also demonstrates pharmaceutical value due to its anti-adhesion, antiviral, and anti-cancer properties, and plays a vital role in red stabilization and prevent blood component aggregation by its negative charge and hydrophilicity. SA also affects fertilization and plays an important role in biological recognition and diseases (2).

SA is very abundant in human milk, as ~70–83% of all SA are bound to human milk oligosaccharides (HMOs), 14–28% are bound to glycoproteins, and 0.2–0.4% to glycolipids, whereas the free form of SA is only 2–3% in human milk (6, 7). Recent studies showed that HMOs play an essential role in regulating neonatal intestinal microecology. Among these HMOs, ~10–30% of oligosaccharides are sialylated (6, 8). In addition to HMOs, sialylated glycoproteins and glycolipids in milk are also abundant and critical to neonatal health (6), they were found regulate intestinal microbial ecosystem and prevent development of neonatal diseases by promoting probiotic growth and metabolism, inhibiting pathogen adhesion, inducing intestinal epithelial differentiation, promoting intestinal maturation, and optimizing immune function (6, 7, 9–11). Some recent studies also support the promoting effects of sialylated milk components on neonatal bone and brain development (12, 13).

Given the important role of intestinal gut microbes in infant nutrition and health, as well as the development of immune system, this article reviews the research progress in the regulation of sialylated milk oligosaccharides and glycoconjugates on neonatal gut microbiota and health. Although some of their effects on neonatal health have been reviewed in the past, the content has been incomplete and most of the discussion has focused on sialylated oligosaccharides. Here we focused on the regulation of sialylated milk components on neonatal gut microbiota, and updated new related researches for better understanding the important role of sialylated milk components in improving neonatal physiology and health through regulating neonatal gut microbiota.

## The Sialylated Components in Milk

### The Structures of Sialylated Milk Components

#### The Structures of Sialylated Milk Oligosaccharides

**Figure 2:**
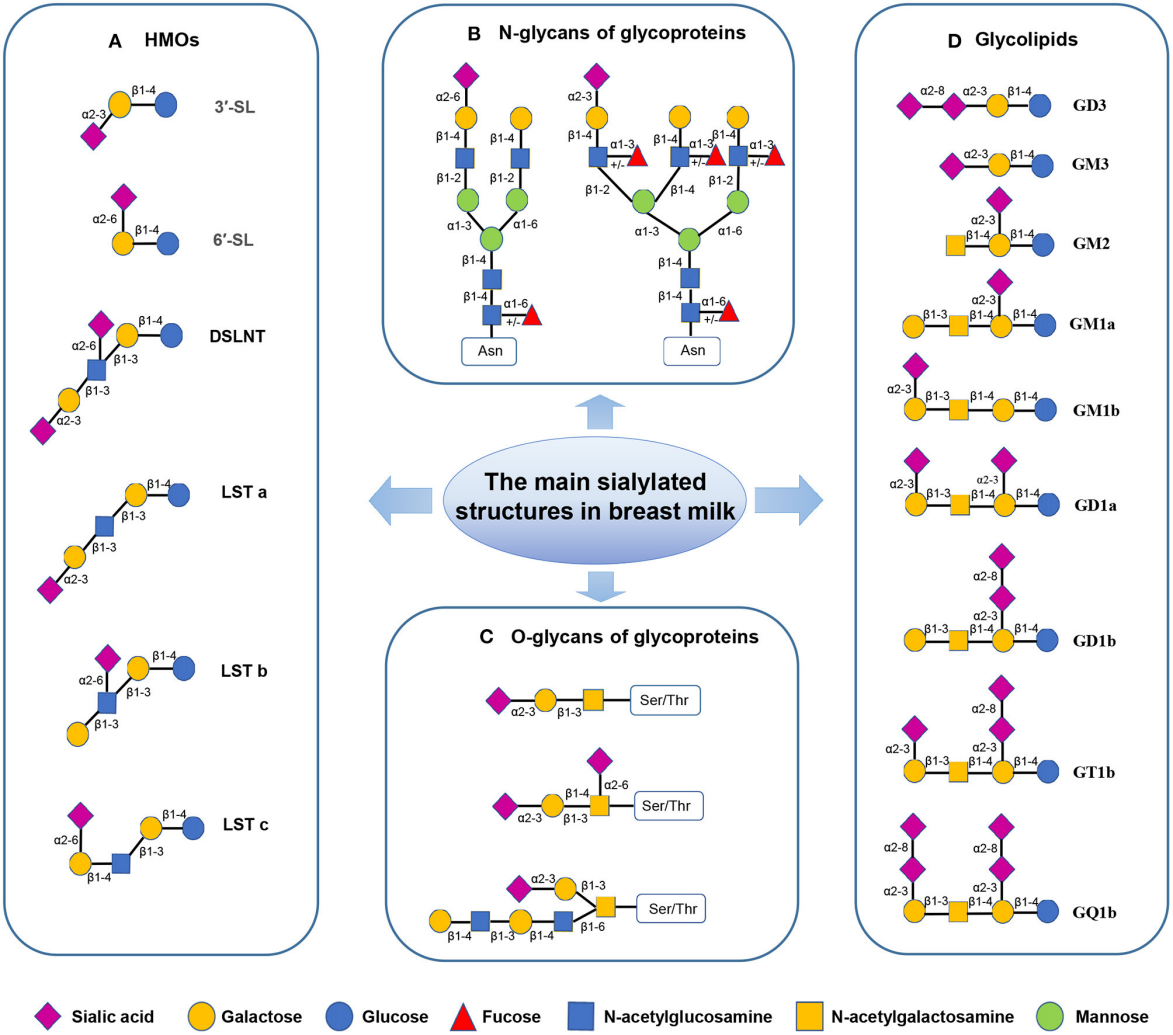
The sialylated structures in breast milk (7, 17–20). (Monosaccharide key is shown at the bottom of the figure.) **(A)** Sialylated HMOs: lactose can be fucosylated or sialylated in different linkages to generate trisaccharides, such as 3′-SL and 6′-SL; The elongated chains can be sialylated by α2–3 or α2–6 linkages at the terminal positions forming structural isomers, such as DSLNT and LST. **(B)** Sialylated N-glycans of glycoproteins: The two most common structures in human milk N-glycome are presented; Asn-asparagine. **(C)** Sialylated O-glycans of glycoproteins; Ser-serine, Thr-threonine. **(D)** Gangliosides: In Svennerholm nomenclature, the capital letter “G” is used to denote the core saccharide of the “Ganglio” series, followed by the capital letters (M, D, T, Q) to indicate the total number of sialic acids. The following *Arabic numerals* (1–4) denote the length of the neutral core (21).

Human milk oligosaccharide is the most common solid component in breast milk, which is the third most abundant breast milk component, after lactose and lipids (14). It comprises D-glucose (Glc), D-galactose (Gal), N-acetylglucosamine (GlcNAc), L-fucose (Fuc), and Neu5Ac. So far, more than 200 HMO structures have been identified. These HMOs range from 3 to 32 monosaccharides in size and indigestible by the host (15). HMOs are divided into acidic and neutral oligosaccharides. Acidic oligosaccharides contain SA molecules, while neutral oligosaccharides do not (7). Neu5Ac is the only form of SA found in human milk, while milk of other mammals may also contain N-glycolylneuraminic acid (Neu5Gc)-bound oligosaccharides (16). Sialylated HMOs are mainly composed of 3′-sialyllactose (3′-SL), 6′-sialyllactose (6′-SL), LS-tetra-saccharide a (LSTa), LS-tetra-saccharide b (LSTb), LS-tera-saccharide c (LSTc), and disialyllacto-N-tetraose (DSLNT) (17, 18) (Figure 2A), and the concentration of them is about 1,000–3,300 mg/L in the colostrum and droped to about 135–2,150 mg/L in the matured human milk (Table 1). Among all sialylated HMOs, the content of sialyllactose (SL) is the highest, with an estimated concentration of 170–500 μg/mL for 6′-SL in mature breast milk (6, 22–24). SL play important roles in neonatal gut maturation, prevention of pathogen invasion, immune regulation, and prebiotic function (33).

**Table 1:** The concentrations of sialylated oligosaccharides and glycoconjugates in human milk.

**Structures**	**Concentration in human milk (mg/L)**			**References**
	**Colostrum**	**Transitional**	**Mature**	
Total Sialylated HMO	1,000–3,300	n.a.	135–2,150	(6)
6′-SL	250–1,300	n.a.	170–500	(6, 22–24)
3′-SL	90–350	n.a.	170 - 500	(6, 22–24)
DSLNT	78–2,500^*^			(25)
LSTa/b	n.a.	104 ± 46	31 ± 25	(26)
LSTc	n.a.	488 ± 224	11 ± 8	(26)
6′-SLN	n.a.	15 ± 15	5 ± 1	(26)
Total protein	8,000–10,000	n.a.	7,000–8,000	(27)
LF	9,700	n.a.	2,000–3,000	(28)
SIgA	6.51–1,359.61^*^			(29)
Mucins	729 ± 75^*^			(30)
κ-casein	450 ± 80	n.a.	1,050 ± 280	(31)
OPN	178.0 ± 17.9	n.a.	48.3 ± 10.2	(32)
BSSL	100–200^*^			(30)
Total GA	n.a.	21.18 ± 11.46	20.18 ± 9.75	(26)
GM3	n.a.	9.47 ± 8.37	18.62 ± 9.69	(26)
GD3	n.a.	11.71 ± 9.46	1.57 ± 2.24	(26)

*n.a. = Data not available*.

* *The stages of breast milk samples were not indicated*.

*Values (mg/L) are means ± standard deviation*.

#### The Structures of Sialylated Milk Glycoconjugates

##### Sialylated Glycoproteins in Milk

Researchers estimated that up to 70% of human milk proteins are glycosylated, and these proteins help shape the intestines and immune system of developing infants (34). The most abundant human milk glycoproteins include lactoferrin (LF, 17% of total protein), α-lactalbumin (17%), secretory immunoglobulin A (sIgA, 11%), and κ-casein (9%). The antipathogenic effects of human milk glycoproteins, such as LF, κ-casein, sIgA can be partly attributed to their sialylated glycan moieties (27).

##### Lactoferrin (LF)

LF is a highly sialylated iron-binding glycoprotein, which is the most abundant glycoprotein (80 KDa) in human milk, accounting for 1/4 of the total protein in human milk (35). Its concentration is highest in colostrum approximately 9.7 g/L and declines to 2–3 g/L in humans' mature milk (Table 1). LF concentration in mature bovine milk is 0.03 g/L−0.1 g/L, about 1/10 of that in human milk (28). Human lactoferrin (HLF) exhibits three potential N-glycosylation sites (Asn137, Asn478, and Asn623) (36, 37). In contrast, bovine lactoferrin (BLF) exhibits five (Asn233, Asn281, Asn368, Asn476, and Asn545). Alternatively, murine LF exhibits only one potential N-glycosylation site: Asn476. Glycans attached through N-glycosidic bonds may contribute to the functional activity of LF (38, 39). Researchers reported that HLF possesses multiple sialylated or fucosylated N-glycans (Figure 2B), which exhibit highly branched complexes or hybrid or both (40).

##### κ-casein

κ-casein is another major glycoprotein in human milk, especially rich in the matured human milk (Table 1). It possesses seven O-glycosylation sites at its C-terminal (27). Its carbohydrate content is 40–60%, while bovine κ-casein carbohydrates are only ~10% (41). Compared with bovine κ-casein, the glycan part of human κ-casein is rich in SA (6).

##### Secretory Immunoglobulin A (sIgA)

SIgA is a heavily glycosylated protein, built from “fucose, galactose, SA, and mannose residues.” Moreover, monosaccharides, namely, GlcNAc is missing (42, 43). SIgA exhibits both N-linked and O-linked glycans (Figure 2C). More than 75% of the N-glycans in the J chain are sialylated, while <15% of the N-glycans in the H chain are sialylated (43). SIgA accounts for 80–90% of all immunoglobulins in human milk, with the highest concentrations found in premature mothers' colostrum and breast milk (Table 1).

##### Mucin

Mucin is an acidic glycoprotein with high molecular weight, ranging in size from 200 KDa to 2,000 KDa. Lactadherin is a mucin-related sialylated glycoprotein in the milk fat globule membrane (MFGM), which includes five N-linked glycosylation sites.

##### Osteopontin (OPN)

OPN is an acidic glycosylated protein with rich SA (44), which is found in high levels in breast milk (Table 1) but in low levels in milk and infant formula, and is an important immunoactive protein in breast milk. The glycosylation of OPN was dominated by O-glycan. Studies have shown that OPN plays an important role in improving immunity, promoting intestinal health and promoting cognitive development in infancy (45).

##### Bile Salt-Stimulated Lipase (BSSL)

BSSL is a highly glycosylated protein in human milk, with 10 potential O-linked glycosylation sites at the C-terminus of the protein, heavily decorated with carbohydrates including galactose, glucosamine, fucose, galactosamine and SA in molar ratios of 3:2:1:1:0.3, respectively (27). The concentration of BSSL in colostrum of GDM mothers was lower than that of normal mothers. BSSL helps infants digest fat early in life, and its levels are associated with breastfeeding (46). Purification and characterization of recombinant human bile salt-stimulated lipase expressed in milk of transgenic cloned cows (27).

##### Sialylated Glycolipids in Milk

Glycolipids of human milk are divided into neutral glycolipids-without SA and acidic glycolipids-containing SA (such as gangliosides). Gangliosides (GAs) are the most abundant in human milk glycolipids (Table 1) (47–50). Many types of GAs exist in breast milk, such as GD3, GM3, GM2, GM1a, GM1b, GD1a, GD1b, GT1b and GQ1b, among which monosialoganglioside 3 (GM3) and disialoganglioside 3 (GD3) are key (51) (Figure 2D). In breast milk, the content and distribution of GAs change during lactation and vary between individuals. GD3 is the most abundant GA in colostrum, whereas GM3 is the main GA in mature milk (27). Milk GAs seem to prevent the adhesion of pathogens and improved the intestinal ecology of new-borns. GM3 is a receptor analog of intestinal cells, which can resist pathogens, such as enterotoxigenic and EPEC, and it has a protective role against infections (52).

### Factors Affect the Levels of Sialylated Components in Milk

Sialylated oligosaccharides are a crucial component of HMOs, especially in the early stages of breast feeding, as they account for 20–30% of the total HMOs, and keep decreasing along with the extension of lactation process (53). The composition and concentration of HMOs vary among individual mothers and change during different lactation stages (54–56). The variation among different mothers is in part due to the genetic polymorphisms in fucosyltransferase-2 (FUT2) and FUT3, which encode the Secretor and Lewis genes, respectively. The polymorphisms in these genes altered the activities of fucosyltranserase and thus lead to distinct fucosylation patterns of HMOs (54, 55, 57, 58). However, the levels of sialylated HMOs were also found affected by mothers' secretor status. Xu et al. reported a higher content of sialylated HMOs in milk of non-secretor compared to milk of secretor mothers, they found that the sialylation level of secretory mothers is 26% lower than that of non-secretory mothers on the 120th day after birth (56, 59), while others found no difference (60, 61). In a study on the effects of breast milk oligosaccharides on iron and galactose oligosaccharide interventions in Kenyan infants, researchers found no significant anthropometric difference at baseline between infants of secretory and non-secretory mothers (61). Another study on stunted infants in Malawi found that non-secretory mothers of severely stunted infants exhibited lower concentrations of sialylated and fucosylated breast milk HMOs than those of non-secretory mothers of healthy infants (29). In addition to genetic factors, environmental factors such as geographic location and maternal nutritional status may also influence HMO concentration and composition (62–64). Preterm milk is another factor to affect HMOs concentration and composition. Alteration in concentration of sialylated HMOs were found in preterm milk, in particular the concentration of 3′-SL was elevated (65). Interestingly, HMOs were also detected in the serum of pregnant women and with increasing concentrations along with pregnancy (65). In a prospective longitudinal cohort study including 87 overweight or obese women, Jantscher-Krennand et al. (66) found that the sialylated HMOs, including 3′-SL and 3′-sialyllactosamine (3′-SLN), in serum of the pregnant women, were positively associated with fasting glucose level, suggesting that metabolism alterations during pregnancy may also affect HMOs pattern, and this influence may last till lactation to affect the infants. A previous study of our group (67) using mouse models with high-fat diet and streptozotocin-induced gestational diabetes mellitus (GDM) found that, there was a decreasing pattern in the concentration of milk oligosaccharides of GDM mice compared with that of the control mice, but with no significant statistic differences. However, the milk of GDM maternal mice contained significantly higher concentrations of fucosylated and sialylated N-glycans than the control mice. The alteration in milk glycobiome of GDM mice had direct effects on the intestinal microbiome of the offspring, which in turn affected their immune responses. Given this study is based on mouse models, which differs largely from human samples, future studies in the feature of HMOs of GDM mothers, as well as the pattern of glycoconjugates in human milk are urgently needed and will lay a foundation for the development of specific nutritional care for the GDM infants. In addition to HMOs, the levels of milk protein and lipids can also be affected by many maternal factors, including smoking, BMI, birth route, pregnancy weight gain, and energy intake during lactation. For example, Aksan et al. (68) have detected significant correlations between body weight, length, and head circumference, respectively, and OPN levels after one (*r* = 0.442, *p* = < 0.001; *r* = −0.284, *p* = < 0.001; *r* = −0.392, *p* = < 0.001) and 3 months (*r* = 0.501, *p* = < 0.001; *r* = −0.450, *p* = < 0.001; *r* = −0.498, *p* = < 0.001) of lactation.

### Technologies and Methods of Investigating Sialylated Milk Components

The key technologies and methods of investigating sialylated oligosaccharides and glycocomplex in recent years are summarized in Table 2. The most common analytical method used to quantify HMOs were high performance liquid chromatography (HPLC) (69) with fluorescence detection and high-performance anion-exchange chromatography (HPAEC) (70) with pulsed amperometry detection (PAD) (61). Other methods involved LC-MS (liquid chromatography with mass spectrometry)/MRM (multiple reaction monitoring) (71), CE (capillary electrophoresis) (72), NMR (nuclear magnetic resonance) (73), and nano-LC-chip-TOF (time of flight) (74) were also used to precisely detect the concentration and structures of the milk oligosaccharides. But for a long period, detection of acid milk oligosaccharides has been a technical problem. In recent years, Yan et al. (75, 76) developted a new method of solid-phase extraction (SPE) with hydrophilic interaction chromatography (HILIC) followed by mass spectrometry (MS) identification for the analysis of sialylated milk oligosaccharides. Collision-induced dissociation tandem ESI-MS (ESI-CID-MS/MS) is then used for sequence and sialic acid α2-3/α2-6 linkage analysis. For the detection of glycoconjugates in milk, the LC-MS method (43) was generally adopted. For example, the most common analytical methods for N-Glycans or O-Glycans were MS, HPLC, LC-ESI-MS/MS and Nano-LC-chip-TOF (43, 52). The ganglioside in breast milk was normally determined by HPLC-MS (77, 78).

**Table 2:** The key technologies and methods for investigating sialylated oligosaccharides and glycoconjugates in milk.

**Technologies and methods**	**References**
**Milk oligosaccharides**	
High performance liquid chromatography, HPLC	(69)
High-performance anion-exchange chromatography, HPAEC	(70)
Pulsed amperometry detection, HPAE-PAD	(61)
Liquid chromatography with mass spectrometry and multiple reaction monitoring, LC-MS/MS-MRM	(71)
Capillary electrophoresis, CE	(72)
Nuclear magnetic resonance, NMR	(73)
Nano-liquid chromatography-chip-time of flight, Nano-LC-chip-TOF	(74)
Hydrophilic interaction chromatography, ESI-CID-MS/MS	(75, 76)
Collision-induced dissociation tandem ESI-MS, SPE-HILIC-MS	(75, 76)
**Milk glycoconjugates**	
Mass spectrometry, MS	(43)
HPLC	(43)
LC-ESI-MS/MS	(43)
Nano-LC-chip-TOF	(43, 52)
HPLC-MS	(77, 78)

## Effects of Sialylated Milk Components on Neonatal gut Microbiota and Health

### Anti-infection

#### The Anti-infection Function of Sialylated Milk Oligosaccharides

The intestinal epithelium cells are covered by a large number of glycoproteins such as mucins. The glycans on these glycoproteins are major components of the gastrointestinal mucosa, and provide essential nutrients and ligands to induce host signaling to defense the invasion of pathological microorganisms and regulate the commensal microbiota. SA-containing glycans were found ubiquitously expressed by gastrointestinal epithelial cells (79–81). As the infection receptors, specific sugar residues, particularly sulfated or sialylated glycans on the mucosal surface can be recognized by many viruses. HMOs were found to prevent the colonization of viral pathogens through two proposed mechanisms. Firstly, as the structures of HMOs share homology with glycans on epithelial cell surface, they can prevent the early cellular attachment as soluble decoy receptors for virus or pathogens. For example, the sialic acid-α2,6 galactose (SA-α2,6Gal) epitope was found to be a receptor for human influenza virus (79) and the sialic acid-α2,3 galactose (SA-α2,3Gal) is a receptor for coxackievirus A24 (80). Another way is that HMOs bind to epithelial cell surface receptors to block viral adhesions (82). Therefore, supplement of SA-linked HMOs from mother's milk can protect intestinal cells of the infants from many viral infections, including influenza, rotavirus (83) and the respiratory syncytial virus (RSV). For example, an *in vitro* hemagglutination inhibition assessment of 3′-SL and 6′-SL against thirteen avian influenza (AI) viruses showed that 3′-SL can inhibit almost all subtypes of the tested AI viruses, whereas 6′-SL only exhibited anti-virus activity against few strains such as H1N1, H1N2, and H3N2. Further *in vivo* study found that administration of 3′-SL to H9N2-infected chickens resulted in elimination of the virus within 24 h post infection (84). The underlying mechanism is their ability to bind to haemagglutinin (HA) glycoprotein spikes of the influenza virus (85). Another study had proved that the combination of 3′-SL and 6′-SL was more effective than single application of each of them in binding to VP8^*^ in a porcine rotavirus model. SA-containing HMO was also shown to inhibit rotavirus infectivity *in vitro* (86); however, both acidic and neutral HMOs were able to decreased NSP4 replication during acute rotavirus infectionin *situ*. Laucirica et al. (87) demonstrated that sialylated oligosaccharides can reduce the infectivity of human rotavirus, and they were considered as possible components to inhibit cholera toxin. The rabbit intestinal loop method was used to observe the effect of SL on cholera toxin-induced diarrhea. The results showed that SL was related to the inhibitory activity of milk against cholera toxin (88). In addition, application of 3′-SL has been shown to significantly decrease the cytokine level and RSV viral in airway epithelia (89).

Similar to the anti-virus activities, different sialylated HMOs can also selectively inhibit bacterial pathogens adhesion to sialylated receptors on the intestinal epithelium by direct binding (10). For example, sialylated HMOs have a strong inhibitory effect on hemagglutination induced by enterotoxigenic *Escherichia coli* (ETEC) and uropathogenic *Escherichia coli* (UPEC) (90). 3′-SL was found to bind to *Helicobacter pylori* and enteropathogenic *Escherichia coli* (EPEC) to inhibit their adhesion to human intestinal cells (HT-29, Caco-2) (38, 39, 91). Angeloni et al. (91) found that *in vitro*, 3′-SL reduced the expression of sialyltransferases ST3Gal1, ST3Gal2, and ST3Gal4, resulting in reduced glycosylation on the surface of Caco2 cells, thus reducing 50% reduction of EPEC adhesion. Further the study (92) found that 3′-SL also had an inhibitory effect on *Salmonella fyrisby*. 6′-SL has anti-adhesion effect on *Escherichia coli* O119, but on *Salmonella fyris*. And 6-SL has been shown to effectively inhibit pneumocyte invasion *Pseudomonas aeruginosa* strains. Acidic HMO components have an inhibitory effect on pathogens expressing specific fimbrial types, such as *Escherichia coli* expressing P and CFA fimbriae (92). In addition to *Escherichia coli, Streptococcus agalactiae* (Group B Streptococcus, GBS) is another leading cause of invasive bacterial infections in infants. Studies found that HMOs may function as an alternative substrate to modify a GBS component by impairing growth kinetics (93). A recent study demonstrated that sialylated variants of lacto-N-tetraose exert antimicrobial and antibiofilm action against GBS by increasing cellular permeability (94).

#### The Anti-infection Function of Sialylated Milk Glycoconjugates

The major sialylated glycopriteins in milk also possess the ability to inhibit the infection of both pathogenic bacteria and virus. LF is an antibacterial agent with a bacteriostatic effect on the neonatal intestinal mucosa. Its iron-chelating properties can prevent various pathogen growth dependent on iron proliferation (95, 96). The basis of these mechanisms of action is mainly attributed to the protein trunk; however, the N or O-glycans attached on LF protein may play a vital role. For instants, the SA residue on BLF was found to directly bind to Ca^2+^ ions that otherwise seem to stabilize LPS on the outer membrane of bacteria, and the SA portion of HLF can act in a similar manner. In addition, LF can act as a decoy to bind to a variety of microbial pathogens, causing these pathogens to deviate from receptor sites on the surface of host cells (27). LF inhibits the adhesion of intestinal enteric to eukaryotic cell lines (96) and the adhesion inhibition of microorganisms to host cells. It exhibits direct cytotoxicity to bacteria, viruses, and fungi (97). Sialylated glycans of human milk κ-casein was found can inhibit the combination of *Streptococcus mutans* GS-5 to saliva-coated hydroxyapatite (98). After entering the intestine, κ-casein is cleaved by proteases to form glycomacropeptide (GMP), which exhibits antimicrobial properties. Casein GMP is the C-terminal part of κ-casein and locates on the 106–109th amino acid. GMP is present in both human and bovine milk (96, 99). κ-casein exhibits multifaceted protective effects on intestinal infection in infants. Researchers reported (27) that the probiotic effect of GMP may be because it contains Neu5Ac. For example, researchers showed that GMP with SA in bovine κ-casein can inhibit the adhesion of enterohemorrhagic *Escherichia coli* and *Salmonella enteritis* to Caco-2 cells. Further, κ-casein can inhibit pathogens' adhesion to the surface of gut cells in infants. Researchers reported (100) that GMP can inhibit splenocyte proliferation induced by concanavalin A and phytohemagglutinin. After neuraminidase digestion, GMP loses its inhibitory activity on mitogen-induced splenocyte proliferation, indicating that SA is the key to this phenomenon. However, after GMP digestion with trypsin and Streptomyces protease, the inhibitory effect was enhanced, indicating that the peptide chain was also involved. Together, studies suggest that κ-casein is crucial for protecting the infant intestinal tract. O-glycans of sIgA can bind to microorganisms, thereby inhibiting pathogens from adhering to the intestinal epithelium (101). Further, sIgA glycans containing SA can be used as bait to prevent pathogenic bacteria from binding to their glycosylated targets on the intestinal mucosa's surface (30, 101). sIgA can effectively inhibit the adhesion of S-fimbrial *Escherichia coli* by the specific interaction between sialylated N-and O-linked glycans and bacterial adhesins, thus, protecting new-borns from sepsis and meningitis caused by these pathogens (43, 101, 102). Also, the sIgA glycan plays a structural and functional role. For instance, sIgA is resistant to proteolytic digestion in the gut due to its glycan moiety attachment. Therefore, sIgA plays a vital role in protecting new-borns from pathogenic infection and promoting intestinal homeostasis. The antipathogenicity properties of lactadherin in human infants are mainly related to the prevention of rotavirus infection (27, 47). Sialylated glycans of milk mucins can bind to rotavirus and inhibit its replication both *in vitro* and *in vivo* (98). Mucin 1 and 4 are two key mucins identified in human MFGM, which can interact with microorganisms (27). The most researched mechanism is that the SA portion of mucin 1 interacts with pathogens, inhibiting pathogens' ability to bind to their sugar chain receptors on the surface of infant host cells. Therefore, mucin 1 exhibits an essential role in innate immune resistance against invasive microorganisms (30, 47). SA is one of the components of intestinal mucin glycans. When mucin is sialylated in the intestine, its molecular structure is more stable. It cannot be easily degraded by bacteria. In contrast, probiotics can increase the synthesis and secretion of mucin to improve intestinal mucosa's biological barrier function (103). *Ruminococcus gnavus* is human intestinal symbiotic bacteria, which can degrade mucin. Also, *Ruminococcus gnavus* ATCC 29149 binds to gut mucus through SA mediation (104). Presently, few studies exist on sialylated mucin in breast milk; therefore, its effect on infant intestinal microecology needs to be further studied in the future.

### Promoting the Growth of Beneficial Bacteria

#### The Promoting Effects of Sialylated Milk Oligosaccharides on Beneficial gut Bacteria

Studies have found that sialylated HMOs play an important role in promoting the growth of beneficial bacteria. For instance, in an *in vitro* study by Zhuo et al. (10), to evaluate the response of individual bacteria to individual components of HMOS, each of 25 major strains isolated from the human gut microbiota was cultured with individual major fucosylated and other sialylated HMOs components. This allowed for an assessment of the effects of specific HMOs on the growth and metabolites of individual microorganisms. The results showed that supplementation with 6′-SL and 3′-SL promoted the growth of *Bifidobacterium lougum, Bacteriodes vulgatus*, and *Bacteroides thetaiotaomicron*. Among the 25 strains tested, these bacteria showed higher neuraminidase activity and produced a large amount of lactate or short-chain fatty acids (SCFAs) or both, which are beneficial for intestinal health and immune function in infants and young children (10, 105). An *in vivo* study also showed that addition of bovine derived oligosaccharide mixtures rich in 3′-SL and 6′-SL to infant formula led to changing in gut microbiota of infants (106). Animal studies showed that SL can improve intestinal dysbiosis and reduce anxiety through gut-brain axis (107). Supplementation of formula with SLs can also modulate gut-associated microbiota in neonatal pigs, which may have important health benefits for developing newborns (33).

The 6′-SL and 3′-SL in milk are partially hydrolyzed in the acidic conditions of the infant's stomach and the remaining SLs are utilized by intestinal bacteria such as *Bifidobacterium, Lactobacillus* and *Bacteroides vulgatus*. Kiyohara et al. isolated an exo-α-sialidase gene (SiabB2) from *Bifidobacterium bifidum* JCM1254 through expression cloning. Expression of SiabB2 in *Bifidobacterium longum* 105-A enabled this strain to degrade sialylated oligosaccharides present in human milk, suggesting that SiabB2 plays an essential role in the catabolism of sialylated HMOs by *Bifidobacterium bifidum* (108). *Bifidobacterium longum* subsp. *infantis* was found to express a sialidase that cleaves α2-6 and α2-3 linkages to utilize milk sialyloligosaccharides. Two-candidate sialidase encoding genes (NanH1 and NanH2) have been isolated from *Bifidobacterium longum* subsp. *infantis* ATCC15697 (109). Although NanH1 is the first sialidase-encoding gene that located in a cluster of genes that specialize in the catabolism of SA, the sialidase NanH2 from *Bifidobacterium bifidum* strains were found utilize HMO and degrade milk sialyloligosaccharides on their extracellular surface, but not NanH1, which suggested that NanH1 may be active for other sialylated glycans encountered in the infant's gut (109). A recent study of the consumption of HMOs by strains of *Bifidobacterium breve* revealed that all of those tested strains can utilize sialylated lacto-N-tetraose (30). These results indicate that *Bifidobacterium* spp. play an important role in the utilization of sialylated HMOs.

#### The Promoting Effects of Sialylated Milk Glycoconjugates on the Growth of Beneficial Bacteria

Researchers have found that the abundance of *Bifidobacterium* and *Lactobacillus* in feces of breast-fed new-born was significantly correlated with fecal LF level (110). Expression of enzymes such as Endo-β-N-acetylglucosaminidases by infant-associated *Bifidobacterium* spp. was found to help them to release the complex N-glycans from LF, incubation of the bacterium with HLF or BLF led to the induction of genes associated to import and consumption of HMOs, suggesting linked regulatory mechanisms among these glycans. These findings indicate that HLF can promote the growth of beneficial bacteria and regulate intestinal homeostasis in neonates, thereby contributing to the establishment of a healthy intestinal microbiota profile (15, 111–114). Studies of κ-casein revealed that the GMP part of κ-casein can promote the growth of many beneficial microorganisms in the intestinal tract of infants, including *Bifidobacterium* and *Lactobacillus bifidum*, and thus preventing the colonization of pathogens (27). Some researchers also reported that supplementation of GAs in infant formula, with concentrations similar to those in human milk, can improve the intestinal ecology of premature infants by increasing the abundance of *Bifidobacterium* and reducing the content of *Escherichia coli*, suggesting that GAs play a significant role as prebiotics in the infant gut. In addition to these effects, a number of studies have also revealed that specific *Bifidobacteria* are able to catabolize GAs from milk. For example, *Bifidobacterium infantis* and *Bifidobacterium bifidum were found to* utilize the two milk GAs, GD3 and GM3, whereas *Bifidobacterium breve* did not utilize these GAs (52). And the important role of breast milk GAs in the establishment of intestinal *Bifidobacteria* has also been supported by clinical studies (16).

### Promote the Intestinal Maturation and Mucosal Barrier Function of Neonates

#### Effects of Sialylated Milk Oligosaccharides on Intestinal Maturation and Mucosal Barrier Function of Neonates

The intestinal epithelial cells serve as a physical and biochemical barrier that separates the microbiota from the gut epithelium, and this mucosal barrier can as well-facilitate the communication between microbiota and immune system (115). The fermentation products of SLs from gut bacteria, especially from *Bifidobacterium* spp., are mainly lactate and SCFAs (10, 116). These products play important role in serving as nutrients for epithelial cells (117). For example, using adult and infant human epithelial cell lines and fecal batch cultures, Perdijk et al.'s study (11) found that, 6′-SL and 3′-SL can induce epithelial differentiation and wound repair, the effect may correlate with the upregulation of SCFAs production and increased abundance of *Bacteroides, Ruminococcs obeum, F. prausnitizii*.

In additon to in-direct effects on intestinal barriers through microbiota-metabolism, SLs have direct effects on intestinal epithelial cell proliferation and differentiation. As early as 2008, some *in vitro* studies have suggested that acidic milk oligosaccharides may inhibit the proliferation of intestinal epithelial cells and induce differentiation (118, 119). This effect is mediated via activation of the epidermal growth factor receptor (EGFR) by interaction of SLs with the carbohydrate moieties on this receptor (119). In the same year, Kuntz et al. (9) reported that 6′-SL directly affects the cell dynamics and promotes epithelial cell differentiation *in vitro*. A study (120) of donkey milk oligosaccharides (DMOs) found that 3′-SL and 6′-SL are the primary oligosaccharides in DMOs, they induce differentiation, promoted apoptosis and inhibited proliferation of HT-29, Caco-2 and human intestinal epithelial cells in a concentration-dependent manner, suggesting that DMOs promote maturation of intestinal epithelial cells. And theses effect was found associated with activation of the p38 pathway and cell cycle arrest at the G2/M phase. Recently, Yang et al. (121) explored the molecular and cellular mechanisms by SL intervention with intestinal maturation in neonatal piglets. They found that treatment of 3′-SL and 6′-SL to piglets can upregulate Ki-67 expression in ileum crypts, increase the width of ileum crypt, and reduce the incidence and severity of diarrhea. Their results showed that SL intervention upregulated the expression level of the glial-derived neurotrophic factor (GDNF) in the ileumof piglets, it also upregulated the mRNA expression level of ST8Sia IV, which is the key polysialyltransferase in the synthesis of Polysialic acid (PolySia)-NCAM. PolySia mediates binding to GDNF, activates Fyn, and increases the expression level of cAMP responsive element-binding protein (CREB) phosphorylation. GDNF promotes cell proliferation by upregulating the CREB, evidenced by the increase in the number and density of Ki-67 positive cells in the crypt. *In vivo* studies showed that CREB and its binding protein are required for the survival of intestinal stem cells, and that overexpression of CREB promotes cell proliferation. Further, SL intervention can significantly reduce the incidence and severity of diarrhea in early weaning of piglets. These results suggest that SL promotes intestinal maturation of neonatal piglets by up-regulating the synthesis of GDNF, Polysia and CREB interaction pathways. Another recent study by Natividad et al. (122) investigated the effects of six industrially available HMOs (2′-FL, 3′-SL, 6′-SL, LNnT, LNT and DFL) alone and in different combinations on epithelial barrier function by using an *in vitro* model of two intestinal epithelial cell lines, Caco-2 and HT29. The results showed tha the six HMOs blend dose-dependently limited the cytokine-induced FD4 translocation and decrease the epithelial permeability post challenge. Similarly, 3 and 5 HMO blends including 3′-SL and 6′-SL also conferred a significant protection against the challenge, which suggested that different abilities of specific HMOs in regulating the intestinal barrier and support the potential of complementing available HMOs combinations to promote intestinal health and protect against intestinal inflammatory diseases. Another *in vivo* study by Holscher et al. (123) also found that individual and combined treatment with 2′-FL, 3′-SL and 6′-SL inhibited the proliferation of small intestinal cell lines HT-29 and Caco-2Bbe, while they enhanced differentiation of HT-29 and Caco-2Bbe cells. The combination of inhibition of proliferation and induction of differentiation suggests that sialylated HMOs may contribute specifically to the maturation of intestinal epithelial cells (91). Thus, the underlying mechanisms regarding the effects of sialylated HMOs on intestinal epithelial cells need to be further studied. A possible way to explain their effects is the directly activation of G-protein coupled receptor of cells by sialylated HMOs and in tern upregulated the signal transduction pathways downstream (124). Their results showed that one of the pathways by which HMO activates G-protein coupled receptor 35 (GPR35) is through a direct interaction of 6′-SL. GPR35 is a receptor that mediates pain and colitis attenuation. More recently, Tsukahara et al. (125) reported that the use of GPR35 agonists alleviated DSS-induced colitis in mice. They found that GPR35 agonists promoted *in vitro* intestinal epithelial cell migration, which may contribute to damage repair in colitis However, lack of GPR35 leads to a worsening outcome in DSS-induced experimental colitis, suggesting that GPR35 plays an important role in protecting colon inflammation (126). Another study has shown that GPR35 promotes the proliferation of intestinal epithelial cells (127). Therefore, it was proposed that sialylated HMOs may protect neonatal intestinal health by activating GPR35 through the interaction of 6′-SL.

#### Effects of Sialylated Milk Glycoconjugates on Intestinal Maturation and Mucosal Barrier Function of Neonates

It has been found that LF can reduce the abundance of *Escherichia coli* in colon and promote intestinal maturation, which protects piglets from early weaning diarrhea by up-regulated intestinal gene expression of brain-derived neurotrophic factors, ubiquitin carboxy-terminal hydrolase L1, and alkaline phosphatase activity (128). In addition, the formula containing BLF was found enhanced the proliferation, depth and area of jejunal crypt and the expression of β-catenin mRNA in piglets. The increased expression of β-catenin indicated that Wnt signal may partially mediate the stimulating effect of BLF on intestinal cell proliferation. These findings provide evidence that supporting the role of LF in neonatal intestinal growth and maturation (111). OPN is another milk glycocojugates that have been found to confer protection effects on intestinal maturation and mucosal barrier function of neonates. Based on the potential benefits of OPN in early life, the effects of OPN supplementation on growth, body composition, and intestinal transcriptome in raspus monkey pups were investigated by comparing different feeding patterns including breast milk, OPN formula, and regular formula (129). The results showed that although growth was similar in each group, the intestinal gene expression pattern (such as CUX1 and EGFR) was more similar in the breast milk and OPN formulations groups. In addition, studies have found that OPN in milk can promote the differentiation of intestinal epithelial cells (Caco-2), and stimulate intestinal immunity by upregulating the secretion of IL-18 by intestinal epithelial cells (Caco-2) (45). The possible mechanism by which OPN promotes intestinal health may be through changes in the expression of intestinal genes. For example (129), CUX1 is a protein-encoding gene whose expression product can bind to DNA to further regulate gene expression, influence cell morphological changes and differentiation, and also influence cell life cycle. These processes are crucial to the growth and development of the gut.

### Immune Regulation

#### The Regulation of Sialylated Milk Oligosaccharides on Neonatal Immunity

In addition to regulating bacterial growth and inducing intestinal differentiation, acidic HMOs can affect cytokines' production and lymphocyte maturation. Bode et al. (130) found that acidic fraction of HMOs, mainly 3′-SL and 3′-sialyl-3-fucosyllactose (3′-S-3-FL) significantly inhibited leukocyte rolling and adhesion in a concentration-dependent manner, and therefore serve as anti-inflammatory components to contribute to the lower incidence of inflammatory diseases in human milk-fed infants. Another *in vitro* study (73) showed that acidic HMOs affect cytokine production and activation of cord blood derived T cells, which may influence lymphocyte maturation in breast-fed newborns. They were also found affecting the immune balance of Th1/Th2 by inhibiting the Th2 response of atopic patients, thus regulating the specific immune response of postnatal allergens (56, 90, 131).

3′-SL mediates anti-inflammatory properties by enhancing the expression of peptidoglycan recognition protein 3, a pathogen recognition receptor that has been shown *in vitro* to modulate the inflammatory response (6), and therefore reduce the levels of pro-inflammatory cytokines TNF-α and IL-8 mRNA in Caco-2 cells. It has been showed that supplementation of 3′-SL during infancy could affect bacterial colonization of the nouse intestine, and reduce the susceptibility to DSS-induced colitis during adulthood (132). In this model of colitis, the pro-inflammatory effect of 3′-SL was the direct stimulation of dendritic cells in mesenteric lymph nodes through Toll-like receptor 4, resulted in Th1 and Th17 cells expansion and the overproduction of pro-inflammatory cytokines (133). Although 3′-SL has a pro-inflammatory effect in model with DSS-induced colitis, it is considered to have a protective effect in other aspects (6).

Necrotizing enterocolitis (NEC) is a gut inflammatory disorder which is one of the leading causes of mortality and morbidity of preterm infants (134). Recent studies found that 2′-FL and 6′-SL protect the development of NEC in mouse and pig models by inhibiting the Toll-like receptor 4 signaling pathway. Most importantly, these findings suggest that the use of 2′-FL and 6′ -SL, either alone or in combination, may offer new avenues for prevention of this devastating disease (135). In addition to SL, studies found that another acidic HMO, with high structural specificity, namely DSLNT, can reduce NEC in neonatal rats, and its effect depends on the presence of two SAs (136). However, because the rat model exhibits its limitations, the need to confirm whether the results apply to human infants is paramount. If studies confirm the benefits of DSLNT for human infants, it will be an effective supplement that can be used to prevent or treat NEC in formula-fed infants (136). In a newly published cohort study of premature infants, a total of 80 mothers of preterm infants were sampled to measure DSLNT levels and found that the mothers of NEC infants had lower concentrations of DSLNT in their breast milk compared to the control group (137). Further sequencing results of infants' gut microbiota showed that the relative abundance of beneficial bacteria (such as *Bifidobacterium*) was higher in the feces of preterm infants fed with high levels of DSLNT in breast milk, while the relative abundance of harmful bacteria represented by *Enterobacterium* was lower. These results suggest that DSLNT may be one of the nutritional strategies to help prevent NEC in premature infants. Further work is needed to determine whether DSLNT functions by regulating the microbiome or by acting directly on the host, for example by structure-specific receptor-mediated means to alter immune function and reduce inflammation (137).

#### The Regulation of Sialylated Milk Glycoconjugates on Neonatal Immunity

It was found that LF intervention in gilts can improve serum IgA and sIgA levels (36). Clinical trials confirmed that the potential application of BLF may be used to prevent nosocomial sepsis and NEC in premature infants (128). T regulatory cells' (Tregs) level in preterm infants was lower than that in full-term infants, and the level of Tregs increased under LF prevention. Although Treg cells participate in controlling the intestinal immune response of pathogens and strengthen the important role of BLF in controlling intestinal homeostasis (138, 139), however, in a randomized controlled trial of 2,203 infants (112), researchers found that supplementation of LF did not decrease the incidence rate of NEC or infection. In another randomized trial of low-birth-weight preterm infants in Canada (140), still, no clear answer exists to the benefits of BLF in reducing mortality or morbidity in low birth-weight infants. Considering the other beneficial effects of LF, these results of are disappointing; so, further randomized trials and researches that involves larger population of infants should be conducted. In terms of inflammation, LF was found to help reduce of the excessive immune response by blocking various pro-inflammatory cytokines, such as IL-1 β, IL-6, TNF-α, and IL-8, and inhibiting the activity of free radicals (97, 112), which suggested that HLF plays a vital role in balancing the intestinal microbiota and protection from neonatal inflammatory diseases.

sIgA is a key component in human milk for the regulation of neonatal immunity.The mother's milk provides the only source of sIgA for new-borns in the first month after birth and plays as the first immune defense line of human (15). It was found that the glycans on SIgA in breast milk play an important role in connecting innate and acquired immunity (43). In this study, Royle et al. found that the O-glycan regions on the heavy (H) chains and the SC N-glycans of human sIgA have adhesin-binding glycan epitopes including alpha2-6-linked SAs. These glycan epitopes provide sIgA with further bacteria-binding sites in addition to the four Fab-binding sites, thus enabling it to participate in both innate and adaptive immunity.

In a study measuring the biological activity of cow's milk OPN *in vivo* in a mouse model of OPN knockout (KO) (141), similar to wild-type mother-fed pups, pups fed by KO mothers showed inhibitory effects on LPS-induced TNF-α after bovine OPN supplementation, suggesting anti-inflammatory activity of milk OPN. The results of clinical trials have shown that infants fed with OPN-rich formula had less fever than those fed with standard formula. They had an increased proportion of T cells, and TNF-α levels were more similar to those of breast-fed infants, suggesting that OPN may be involved in the development and maturation of the infant's immune system (142). Breast milk OPN is likely to provide beneficial biological activity for breast-fed infants. The OPN active protein from milk is not readily hydrolyzed by the newborn's gastric juices, so most of the OPN active protein can enter the intestinal tract and perform further functions. Animal studies have shown that OPN active protein in milk can stimulate intestinal development and protect intestinal tract (129, 143). At present, *in vivo* studies on the correlation between OPN active protein and NEC are still very limited. Some scholars conducted a study on preterm piglets: the experimental group fed with OPN active protein formula underwent total parenteral nutrition for 2 days and enteral nutrition for 1.5 days. Results showed that OPN active protein formula feeding significantly reduced the disease severity of NEC in preterm piglets compared with conventional formula feeding (144). This also suggests that OPN active protein may have a potential protective effect on the occurrence of NEC in premature infants.

Since some studies showed that GAs may be involved in the activation of T cells and the differentiation of different lymphocyte subsets, the addition of human breast milk GAs or other sources of GAs to infant formula may play an important role in the proliferation, activation, and differentiation of neonatal immune cells, especially those from the intestinal tract (52). GAs can passively prevent infection in the form of “bait” and actively promote the maturity of the infant immune system by regulating immune cell functions and promoting the secretion of cytokines. In an animal experiment (145), scientists observed that in young mice fed GAs-rich milk powder, two cytokine-secreting cells, lamina propria lymphocytes and Pyle-collecting lymphoid, developed earlier and more in number than those in the control group. Another study (146) showed that young mice feed rich in GAs milk powder demonstrated higher levels of sIgA, a crucial immune factor in early life, suggesting better self-protection. In addition to immune protection, GAs are associated with maintaining immune homeostasis. In the early stage of life, new-borns are in a state of immune imbalance, tending to Th2 immunity; that is, it is easy to exhibit excessive immunity, thereby causing allergic reactions. Presently, GD3 can prevent over-immunity by inhibiting the proliferation of dendritic cell CD4^+^ cells (147).

### Promote Growth: Bone and Muscle Development

#### The Growth Promoting Effects of Sialylated Milk Oligosaccharides on Neonates

In addition to promote the maturation of intestinal barrier, sialylated HMOs were also found able to enhance liver, muscle metabolism by increasing nutrient utilization in undernourished infants (12, 148). A study in two Malawian birth cohorts revealed that the sialylated HMOs are significantly less abundant in milk (6-month-postpartum) of those Malawian mothers with severely stunted infants (148). This study also found that the sialylated bovine milk oligosaccharides (S-BMO) can promote the augmentation of lean body mass gain of infants in a microbiota-dependent manner. Supplementation of S-BMOs to neonatal mice or gnotobiotic piglets enhanced their ability to utilize nutrients for anabolism, resulted in alteration of the bone morphology and metabolism of liver, muscle, and brain. Another study using mouse models revealed that 3′-SL can inhibit the degradation of cartilage, and upregulate the CO12a1 production to promote cartilage regeneration, which protects against osteoarthritic development in mice (149). By adding purified S-BMO with structures similar to those in human milk to the diet of young germ-free mice that were colonized with cultured bacterial strains from a 6-mo-old stunted infant, Cowardin et al. (12) found an increased femoral trabecular bone volume and cortical thickness, reduced osteoclasts and their bone marrow progenitors, and altered regulators of osteoclastogenesis and mediators of Th2 responses. Compare with the control mice, the S-BMO treated mice exhibited microbiota-dependent increase in cecal levels of succinate and in turn activated the tuft cell signaling pathway that linked to Th2 immune responses. A prominent fucosylated HMO, 2′-FL, failed to elicit these changes in bone biology, highlighting the structural specificity of the S-BMO effects. A mouse model study of collagen-induced arthritis showed that, 3′-SL could reduce the severity and incidence of arthritis, inhibit the formation of synovitis and pannus, and suppress cartilage destruction, which suggested that 3′-SL can serve as an inhibitor of p65 phosphorylation to ameliorate the progression of experimental rheumatoid arthritis (150).

#### The Growth Promoting Effects of Sialylated Milk Glycoconjugates on Neonates

Studies in infants and animals have proved that milk proteins significantly influence the growth and body composition of neonates (151). Human milk immunomodulatory proteins including LF and sIgA were found time-dependently and differentially associate with development of infant lean mass and adiposity during first 1 year of lactation (152). A rat study by Shama et al. (153) showed that treatment of a human milk-based protein concentrate contained 101 ± 6 g protein/kg in total and 5 ± 1 g lactoferrin/kg of milk solids supported the growth of weanling rats, suggested its potential use for preterm infants. Wu et al. (154) demonstrated that the formula containing hydrolyzed whey protein (hydrolyzed whey/intact casein = 63/37), could support the normal growth of healthy term infants, to a comparable extent to that of breast-fed infants during the first 3 months of life. Gridneva et al. investigated the relationships between infant/maternal body composition and human milk casein, whey and total protein during the first 12 months of lactation (155). Their results showed a differential effect of human milk casein on development of infant body composition during the first year of life, which suggested its potential application in improving outcome for the infants through interventions. Mucin is another source of a microbiological growth factor present in human milk, in the year of 1953, study of Tomarelli et al. (156) had proved that when fed with a basal diet of a composition including mucin approximating that of human milk resulted in increased growth of weanling rats. Interestingly, some studies suggested that milk components such as LF and OPN can form as a complex, which showed increased bioactivities that may possibly improve outcomes in formula-fed infants (157). BSSL in breast milk was also found to facilitate the ability of digestion and absorption of milk fat and promotes growth of small for gestational age preterm infants (158, 159).

### Promote Brain Development and Cognition

#### Effects of Sialylated Milk Oligosaccharides on Brain and Cognition Development of Neonates

The neural cell membranes contain 20 times more SA than other types of membranes, suggesting that SA plays an important role in neural structure (160, 161). Therefore, whether SL has an effective role in promoting neurodevelopment by altering the concentration of important brain metabolites and neurotransmitters attracted many research attentions. In the year of 2016, Jacobi et al. (33) found that both 3′-SL and 6′-SL could increase the ganglisoside (GA) bound SA in the corpus callosum and cerebellum of piglets. And they can also enhance T-maze performance, increased expression level of mRNA glial fibrillary acidic protein gene encoding, and as well as the expression level of myelin basic protein and myelin-associated glycoprotein in piglets (121). In the year of 2019 (161), Wang et al. found a significant increasing effects of SL supplementation on the absolute levels of myo-inositol (mIns) and glutamate + glutamine (Glx) in piglets. They also detected significant positive correlations of brain N-acetylaspartate (NAA), total NAA, mIns, total choline, total creatine, scyllo-Inositol (SI) and glutathione with total white matter volume; Glu and SI with whole brain volume; and SI with whole brain weight respectively. Sialyllactosamine (SLN) and 3′-SL intake were closely correlated with the levels of brain Glu, mlns and Glx in the treatment groups only. This study provided *in vivo* evidences that sialylated milk oligosaccharides can affect neurotransmitters and alter the brain metabolites in piglets. In a recent study, Hauser et al. (13) explored the long-term consequences of selectively depriving mice of specific sialylated HMO during lactation using a gene knockout (KO) mouse model lacking 6′-SL synthesis-related genes. The study found that 6′-SL in breast milk is essential for cognitive development. When observing the microflora function, researchers found that the number of KEGG pathways that changed in St6Gal1 KO mice during lactation was much greater than that in adult mice. Thus, suggesting that the composition of St6Gal1 KO demonstrates a strong impact on the establishment of intestinal microbiota in the early stage of life, especially in weaning from a milk-based diet to a solid diet. However, further research is needed to clarify these aspects. Given the emerging “gut brain axis” and the important role of SL intervention in neonatal intestinal health and disease prevention, future studies will need to assess more precise and deeper molecular mechanisms (124). In addition, study in mice (107) revealed that supplementation of 6′-SL or 3′-SL can release anxiety during stressor tests, and prevent the gut dysbiosis resulting from stress, and help to maintaine normal numbers of doublecortin (DCX) ^+^ immature neurons. The above results suggested the important roles of SLs in ameliorating behavioral responses during stressor exposure through effects on the microbiota dependent gut-brain axis.

#### Effects of Sialylated Milk Glycoconjugates on Brain and Cognition Development of Neonates

Emerging research in humans, rodents and piglets suggest that LF and other glycoproteins in milk may play unique roles in brain development and cognitive functions of infants (35, 162). For example, study of Chen et al. (35) in postnatal peglets found that LF supplementation can promote early neurodevelopment and cognition by upregulating the brain-derived neurotrophin factor (BDNF) signaling pathway and polysialylation. This study suggested that, as a SA-rich milk glycoprotein, the sialylated gloycans on LF may contributed significantly to this effect. A randomized, controlled trial carried by Li et al. (163) showed that infants receiving formula supplementated with bovine MFGM and lactoferrin for 1 year accelerated the neurodevelopmental profile and improved language subcategories at day 545. Oh et al. (164) studied the effects of glycated milk casein (Gc) fermented with *Lactobacillus rhamnosus* 4B15 (FGc) on the intestinal microbiota and physiological and behavioral properties in mice under chronic stress, and their results strongly suggested the protective effects of FGc targeting of intestinal microbiota for abnormal brain activity, which is consistent with the view that FGc plays an important role in regulating stress-related gut-brain axis disorders. Milk OPN was also found to increase the brain myelination and cognitive development in mice (165). In addition to glycoproteins, glycolipids in milk also contribute to the development of brain and congnition, as complex lipids are important constituents of the central nervous system. Studies have shown that supplementation with complex milk lipids (CML) in pregnancy may increase the level of fetal gangliosides (GA), with the potential to improve cognitive outcomes (166). For example, Liu et al. (167) have reported that early supplementation of phospholipids and gangliosides affects brain and cognitive development in neonatal piglets. A double-blind, randomized, controlled, parallel group clinical trial in which infants received the treatment or control product from 2 to 8 weeks of age until 24 weeks of age. Ganglioside supplementation using complex milk lipids significantly increased the scores for Hand and Eye coordination IQ, Performance IQ and General IQ (168).

## Future Perspectives

**Figure 3:**
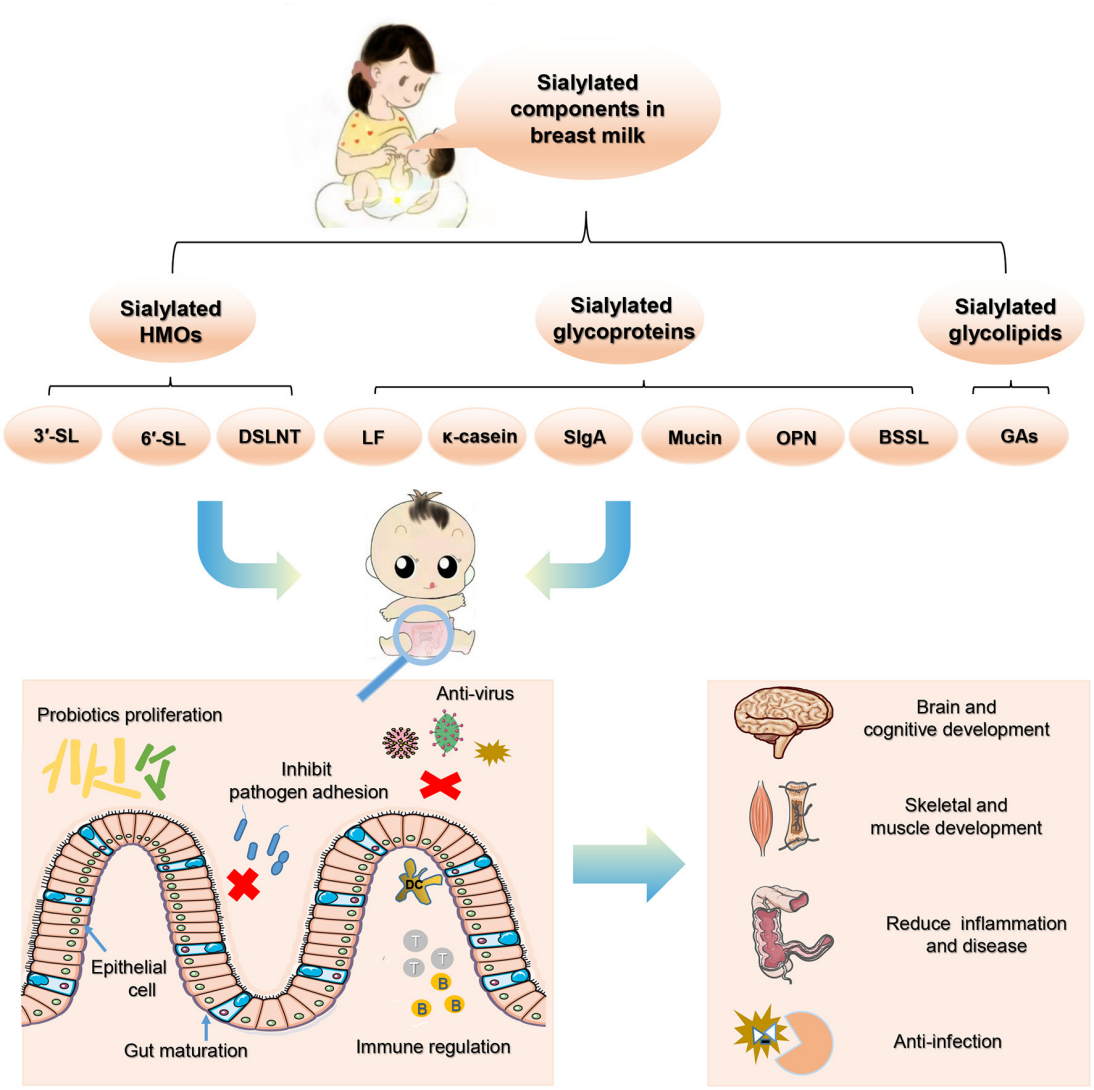
The beneficial effects of sialylated milk components on neonatal health.

To sum up, sialylated milk components exert crucial probiotic and immunomodulatory effects on infant's gut. They play an important role in maintaining colonization resistance, inducing intestinal cell differentiation, promoting intestinal maturation, reducing inflammation, and promoting neonatal growth, all of which are considered to demonstrate significant health benefits to new-borns (Figure 3). As sialylated milk oligosaccharides and glycans exhibit local biological activities in the intestines to protect infants, a healthy establishment of the intestinal microbiota plays a key intermediate role to broaden the effects of sialylated milk components. However, most of these studies are based on animal experiments and *in vitro* studies, and in particular, the function of the sialylated glycan parts on milk glycoproteins and glycolipids on infants remain largely unknown. More supporting evidence of the beneficial effects of sialylated milk oligosaccharides and glycans on infant health will greatly promote the development and utilization of SA-containing microecological preparations. Their application to milk formula, healthy maternal and infant food, and medical purposes holds great potential.

## Author Contributions

YW and XZ wrote a draft version of the manuscript and drew figures. ML, XL, and BR contributed to the design of the review and did the literature search. NZ, WL, JYu, and JYa polished the manuscript and improved the English quality of the manuscript. ML contributed to revised the manuscript and were in charge of the final version of the manuscript. All authors were involved in the conception, preparation of the manuscript, and the final version of the manuscript has been read and approved by all the authors before its submission.

## Funding

This research was funded by the National Natural Science Foundation of China (Nos. 31900920 and 22074143) and the Dalian Science and Technology Innovation Project (2020JJ27SN068). This work was also supported by Liaoning Provincial Program for Top Discipline of Basic Medical Sciences, China.

## Conflict of Interest

XZ and NZ are employed by By-Health Co. Ltd. The remaining authors declare that the research was conducted in the absence of any commercial or financial relationships that could be construed as a potential conflict of interest.

## Publisher's Note

All claims expressed in this article are solely those of the authors and do not necessarily represent those of their affiliated organizations, or those of the publisher, the editors and the reviewers. Any product that may be evaluated in this article, or claim that may be made by its manufacturer, is not guaranteed or endorsed by the publisher.

## Abbreviations

SA: Sialic acid
Neu5Ac: N-acetylneuraminic acid
HMOs: human milk oligosaccharides
Glc: glucose
Gal: galactose
GlcNAc: N-acetylglucosamine
Fuc: fucose
SL: sialyllactose
3′-SL: 3′-sialyllactose
6′-SL: 6′-sialyllactose
LSTa: LS-tetra-saccharide a
LSTb: LS-tetra-saccharide b
LSTc: LS-tera-saccharide c
DSLNT: disialyllacto-N-tetraose
SLN: sialyllactosamine
3'-S-3-FL: 3'-sialyl-3-fucosyllactose
FUT2: fucosyltransferase-2
GDM: gestational diabetes mellitus
RSV: respiratory syncytial virus
AI: avian influenza
GBS: Group B Streptococcus
SCFAs: short chain fatty acids
SiabB2: exo-α-sialidase gene
NanH1 and NanH2: sialidase encoding genes
DMOs: donkey milk oligosaccharides
EGFR: epidermal growth factor receptor
EGF: epidermal growth factor
GDNF: glial-derived neurotrophic factor
PolySia: Polysialic acid
CREB: cAMP responsive element-binding protein
GPR35: G-protein coupled receptor 35
S-BMO: sialylated bovine milk oligosaccharides
mIns: myo-inositol
Glx: glutamate + glutamine
NAA: N-acetylaspartate
SI: scyllo-Inositol
KO: knockout
NEC: necrotizing enterocolitis
ETEC: enterotoxigenic Escherichia coli
EPEC: enteropathogenic Escherichia coli
UPEC: uropathogenic Escherichia coli
LF: lactoferrin
sIgA: secretory immunoglobulin A
HLF: human lactoferrin
BLF: bovine lactoferrin
Tregs: T regulatory cells
GMP: glycomacropeptide
OPN: Osteopontin
BSSL: Bile salt-stimulated lipase
MFGM: milk fat globule membrane
GAs: gangliosides
GM3: monosialoganglioside 3
GD3: disialoganglioside 3
HPLC: high performance liquid chromatography
HPAEC: high-performance anion-exchange chromatography
PAD: pulsed amperometry detection
LC-MS: liquid chromatography with mass spectrometry
MRM: multiple reaction monitoring
CE: capillary electrophoresis
NMR: nuclear magnetic resonance
nano-LC-chip-TOF: nano-liquid chromatography-chip-time of flight
HILIC: hydrophilic interaction chromatography
MS: mass spectrometry
SPE: solid-phase extraction
ESI-CID-MS/MS: Collision-induced dissociation tandem ESI-MS.

## References

[1] Schauer R. Achievements and challenges of sialic acid research. Glycoconj J. (2000) 17:485–99. 10.1023/A:1011062223612 11421344 PMC7087979

[2] Varki A. Sialic acids in human health and disease. Trends Mol Med. (2008) 14:351–60. 10.1016/j.molmed.2008.06.002 18606570 PMC2553044

[3] Schauer R Kamerling JP. Exploration of the sialic acid world. Adv Carbohydr Chem Biochem. (2018) 75:1–213. 10.1016/bs.accb.2018.09.001 30509400 PMC7112061

[4] Kooner AS Yu H Chen X. Synthesis of N-Glycolylneuraminic acid (Neu5Gc) and its glycosides. Front Immunol. (2019) 10:2004. 10.3389/fimmu.2019.02004 31555264 PMC6724515

[5] van Karnebeek CD Bonafé L Wen XY Tarailo-Graovac M Balzano S Royer-Bertrand B. NANS-mediated synthesis of sialic acid is required for brain and skeletal development. Nat Genet. (2016) 48:777–84. 10.1038/ng.3578 27213289

[6] ten Bruggencate SJ Bovee-Oudenhoven IM Feitsma AL van Hoffen E Schoterman MH. Functional role and mechanisms of sialyllactose and other sialylated milk oligosaccharides. Nutr Rev. (2014) 72:377–89. 10.1111/nure.12106 24828428

[7] Lis-Kuberka J Orczyk-Pawiłowicz M. Sialylated oligosaccharides and glycoconjugates of human milk. The impact on infant and newborn protection, development and well-being. Nutrients. (2019) 11:306. 10.3390/nu11020306 30717166 PMC6413137

[8] Ninonuevo MR Park Y Yin H Zhang J Ward RE Clowers BH. A strategy for annotating the human milk glycome. J Agric Food Chem. (2006) 54:7471–80. 10.1021/jf0615810 17002410

[9] Kuntz S Rudloff S Kunz C. Oligosaccharides from human milk influence growth-related characteristics of intestinally transformed and non-transformed intestinal cells. Br J Nutr. (2008) 99:462–71. 10.1017/S0007114507824068 17925055

[10] Yu ZT Chen C Newburg DS. Utilization of major fucosylated and sialylated human milk oligosaccharides by isolated human gut microbes. Glycobiology. (2013) 23:1281–92. 10.1093/glycob/cwt065 24013960 PMC3796377

[11] Perdijk O van Baarlen P Fernandez-Gutierrez MM van den Brink E Schuren FHJ Brugman S. Sialyllactose and galactooligosaccharides promote epithelial barrier functioning and distinctly modulate microbiota composition and short chain fatty acid production *in vitro*. Front Immunol. (2019) 10:94. 10.3389/fimmu.2019.00094 30809221 PMC6380229

[12] Cowardin CA Ahern PP Kung VL Hibberd MC Cheng J Guruge JL. Mechanisms by which sialylated milk oligosaccharides impact bone biology in a gnotobiotic mouse model of infant undernutrition. Proc Natl Acad Sci USA. (2019) 116:11988–96. 10.1073/pnas.1821770116 31138692 PMC6575181

[13] Hauser J Pisa E Arias Vav́squez A Tomasi F Traversa A Chiodi V. Sialylated human milk oligosaccharides program cognitive development through a non-genomic transmission mode. Mol Psychiatry. (2021) 26:2854–71. 10.1038/s41380-021-01054-9 33664475 PMC8505264

[14] Plaza-Díaz J Fontana L Gil A. Human milk oligosaccharides and immune system development. Nutrients. (2018) 10:1038. 10.3390/nu10081038 30096792 PMC6116142

[15] Thai JD Gregory KE. Bioactive factors in human breast milk attenuate intestinal inflammation during early life. Nutrients. (2020) 12:581. 10.3390/nu12020581 32102231 PMC7071406

[16] Zúñiga M Monedero V Yebra MJ. Utilization of host-derived glycans by intestinal lactobacillus and bifidobacterium species. Front Microbiol. (2018) 9:1917. 10.3389/fmicb.2018.01917 30177920 PMC6109692

[17] Bode L. Human milk oligosaccharides: every baby needs a sugar mama. Glycobiology. (2012) 22:1147–62. 10.1093/glycob/cws074 22513036 PMC3406618

[18] Zhang X Liu Y Liu L Li J Du G Chen J. Microbial production of sialic acid and sialylated human milk oligosaccharides: advances and perspectives. Biotechnol Adv. (2019) 37:787–800. 10.1016/j.biotechadv.2019.04.011 31028787

[19] Smilowitz JT Lebrilla CB Mills DA German JB Freeman SL. Breast milk oligosaccharides: structure-function relationships in the neonate. Annu Rev Nutr. (2014) 34:143–69. 10.1146/annurev-nutr-071813-105721 24850388 PMC4348064

[20] Triantis V Bode L van Neerven RJJ. Immunological effects of human milk oligosaccharides. Front Pediatr. (2018) 6:190. 10.3389/fped.2018.00190 30013961 PMC6036705

[21] Schnaar RL. The biology of gangliosides. Adv Carbohydr Chem Biochem. (2019) 76:113–48. 10.1016/bs.accb.2018.09.002 30851743

[22] Seppo AE Autran CA Bode L Järvinen KM. Human milk oligosaccharides and development of cow's milk allergy in infants. J Allergy Clin Immunol. (2017) 139:708–11.e705. 10.1016/j.jaci.2016.08.031 27702672 PMC5303147

[23] Hobbs M Jahan M Ghorashi SA Wang B. Current perspective of sialylated milk oligosaccharides in mammalian milk: implications for brain and gut health of newborns. Foods. (2021) 21;10:473. 10.3390/foods10020473 33669968 PMC7924844

[24] Parschat K Melsaether C Jäpelt KR Jennewein S. Clinical evaluation of 16-week supplementation with 5HMO-Mix in healthy-term human infants to determine tolerability, safety, and effect on growth. Nutrients. (2021) 13:2871. 10.3390/nu13082871 34445031 PMC8401119

[25] Bao Y Newburg DS. Capillary electrophoresis of acidic oligosaccharides from human milk. Electrophoresis. (2008) 29:2508–15. 10.1002/elps.200700873 18512675

[26] McJarrow P Radwan H Ma L MacGibbon AKH Hashim M Hasan H. Human milk oligosaccharide, phospholipid, and ganglioside concentrations in breast milk from United Arab emirates mothers: results from the MISC cohort. Nutrients. (2019) 11:2400. 10.3390/nu11102400 31597293 PMC6835464

[27] Peterson R Cheah WY Grinyer J Packer N. Glycoconjugates in human milk: protecting infants from disease. Glycobiology. (2013) 23:1425–38. 10.1093/glycob/cwt072 24000281

[28] Jahan M Kracht S Ho Y Haque Z Bhattachatyya BN Wynn PC. Dietary lactoferrin supplementation to gilts during gestation and lactation improves pig production and immunity. PLoS ONE. (2017) 12:e0185817. 10.1371/journal.pone.0185817 29023467 PMC5638254

[29] Hogendorf A Stańczyk-Przyłuska A Sieniwicz-Luzeńczyk K Wiszniewska M Arendarczyk J Banasik M. Is there any association between secretory IgA and lactoferrin concentration in mature human milk and food allergy in breastfed children. Med Wieku Rozwoj. (2013) 17:47–52. 23749695

[30] Liu B Newburg DS. Human milk glycoproteins protect infants against human pathogens. Breastfeed Med. (2013) 8:354–62. 10.1089/bfm.2013.0016 23697737 PMC3725943

[31] JLiao Y Weber D Xu W Durbin-Johnson BP Phinney BS Lönnerdal B. Absolute quantification of human milk caseins and the whey/casein ratio during the first year of lactation. J Proteome Res. (2017) 16:4113–21. 10.1021/acs.jproteome.7b00486 28925267

[32] Jiang R Lönnerdal B. Osteopontin in human milk and infant formula affects infant plasma osteopontin concentrations. Pediatr Res. (2019) 85:502–5. 10.1038/s41390-018-0271-x 30636771

[33] Jacobi SK Yatsunenko T Li D Dasgupta S Yu RK Berg BM. Dietary isomers of sialyllactose increase ganglioside sialic acid concentrations in the corpus callosum and cerebellum and modulate the colonic microbiota of formula-fed piglets. J Nutr. (2016) 146:200–8. 10.3945/jn.115.220152 26701794

[34] Zhu J Dingess KA. The functional power of the human milk proteome. Nutrients. (2019) 11:1834. 10.3390/nu11081834 31398857 PMC6723708

[35] Chen Y Zheng Z Zhu X Shi Y Tian D Zhao F. Lactoferrin promotes early neurodevelopment and cognition in postnatal piglets by upregulating the BDNF signaling pathway and polysialylation. Mol Neurobiol. (2015) 52:256–69. 10.1007/s12035-014-8856-9 25146846 PMC4510916

[36] Albar AH Almehdar HA Uversky VN Redwan EM. Structural heterogeneity and multifunctionality of lactoferrin. Curr Protein Pept Sci. (2014) 15:778–97. 10.2174/1389203715666140919124530 25245670

[37] O'Riordan N Kane M Joshi L Hickey RM. Structural and functional characteristics of bovine milk protein glycosylation. Glycobiology. (2014) 24:220–36. 10.1093/glycob/cwt162 24398766

[38] Chaturvedi G Tewari R Mrigank Agnihotri N Vishwakarma RA Ganguly NK. Inhibition of helicobacter pylori adherence by a peptide derived from neuraminyl lactose binding adhesin. Mol Cell Biochem. (2001) 228:83–9. 10.1023/A:1013314604403 11855744

[39] Simon PM Goode PL Mobasseri A Zopf D. Inhibition of helicobacter pylori binding to gastrointestinal epithelial cells by sialic acid-containing oligosaccharides. Infect Immun. (1997) 65:750–7. 10.1128/iai.65.2.750-757.1997 9009338 PMC176121

[40] Nwosu CC Aldredge DL Lee H Lerno LA Zivkovic AM German JB. Comparison of the human and bovine milk N-glycome via high-performance microfluidic chip liquid chromatography and tandem mass spectrometry. J Proteome Res. (2012) 11:2912–24. 10.1021/pr300008u 22439776 PMC3345083

[41] Rudloff S Kunz C. Protein and nonprotein nitrogen components in human milk, bovine milk, and infant formula: quantitative and qualitative aspects in infant nutrition. J Pediatr Gastroenterol Nutr. (1997) 24:328–44. 10.1097/00005176-199703000-00017 9138181

[42] Hughes GJ Reason AJ Savoy L Jaton J Frutiger-Hughes S. Carbohydrate moieties in human secretory component. Biochim Biophys Acta. (1999) 143:486–93. 10.1016/S0167-4838(99)00168-5 10556562

[43] Royle L Roos A Harvey DJ Wormald MR van Gijlswijk-Janssen D Redwan el-RM. Secretory IgA N- and O-glycans provide a link between the innate and adaptive immune systems. J Biol Chem. (2003) 278:20140–53. 10.1074/jbc.M301436200 12637583

[44] Nemir M Bhattacharyya D Li X Singh K Mukherjee AB Mukherjee BB. Targeted inhibition of osteopontin expression in the mammary gland causes abnormal morphogenesis and lactation deficiency. J Biol Chem. (2000) 275:969–76. 10.1074/jbc.275.2.969 10625634

[45] Jiang R Lönnerdal B. Effects of milk osteopontin on intestine, neurodevelopment, and immunity. Nestle Nutr Inst Workshop Ser. (2020) 94:152–7. 10.1159/000505067 32172244

[46] Sha L Zhou S Xi Y Li R Li X. The level of bile salt-stimulated lipase in the milk of Chinese women and its association with maternal BMI. J Biomed Res. (2019) 34:122–8. 10.7555/jbr.33.20180107 32305966 PMC7183295

[47] Newburg DS. Glycobiology of human milk. Biochemistry. (2013) 78:771–85. 10.1134/S0006297913070092 24010840

[48] Newburg DS Chaturvedi P. Neutral glycolipids of human and bovine milk. Lipids. (1992) 27:923–7. 10.1007/BF02535874 1491612

[49] Newburg DS Grave G. Recent advances in human milk glycobiology. Pediatr Res. (2014) 75:675–9. 10.1038/pr.2014.24 24522101 PMC4125201

[50] Pan XL Izumi T. Variation of the ganglioside compositions of human milk, cow's milk and infant formulas. Early Hum Dev. (2000) 57:25–31. 10.1016/S0378-3782(99)00051-1 10690709

[51] Nakano T Sugawara M Kawakami H. Sialic acid in human milk: composition and functions. Acta Paediatr Taiwan. (2001) 42:11–17. 11270179

[52] Lee H Garrido D Mills DA Barile D. Hydrolysis of milk gangliosides by infant-gut associated bifidobacteria determined by microfluidic chips and high-resolution mass spectrometry. Electrophoresis. (2014) 35:1742–50. 10.1002/elps.201300653 24519724 PMC4048636

[53] Chaturvedi P Warren CD Altaye M Morrow AL Ruiz-Palacios G Pickering LK. Fucosylated human milk oligosaccharides vary between individuals and over the course of lactation. Glycobiology. (2001) 11:365–72. 10.1093/glycob/11.5.365 11425797

[54] Thurl S Munzert M Henker J Boehm G Müller-Werner B Jelinek J. Variation of human milk oligosaccharides in relation to milk groups and lactational periods. Br J Nutr. (2010) 104:1261–71. 10.1017/S0007114510002072 20522272

[55] Thurl S Henker J Siegel M Tovar K Sawatzki G. Detection of four human milk groups with respect to lewis blood group dependent oligosaccharides. Glycoconj J. (1997) 14:795–9. 10.1023/A:1018529703106 9511984

[56] Xu G Davis JC Goonatilleke E Smilowitz JT German JB Lebrilla CB. Absolute quantitation of human milk oligosaccharides reveals phenotypic variations during lactation. J Nutr. (2017) 147:117–24. 10.3945/jn.116.238279 27798342 PMC5177733

[57] Stahl B Thurl S Henker J Siegel M Finke B Sawatzki G. Detection of four human milk groups with respect to Lewis-blood-group-dependent oligosaccharides by serologic and chromatographic analysis. Adv Exp Med Biol. (2001) 501:299–306. 10.1007/978-1-4615-1371-1_37 11787693

[58] Bai Y Tao J Zhou J Fan Q Liu M Hu Y. Fucosylated human milk oligosaccharides and N-Glycans in the milk of chinese mothers regulate the gut microbiome of their breast-fed infants during different lactation stages. mSystems. (2018) 3:e00206–18. 10.1128/mSystems.00206-18 30637338 PMC6306508

[59] Azad MB Robertson B Atakora F Becker AB Subbarao P Moraes TJ. Human milk oligosaccharide concentrations are associated with multiple fixed and modifiable maternal characteristics, environmental factors, and feeding practices. J Nutr. (2018) 148:1733–42. 10.1093/jn/nxy175 30247646

[60] Totten SM Zivkovic AM Wu S Ngyuen U Freeman SL Ruhaak LR. Comprehensive profiles of human milk oligosaccharides yield highly sensitive and specific markers for determining secretor status in lactating mothers. J Proteome Res. (2012) 11:6124–33. 10.1021/pr300769g 23140396

[61] Paganini D Uyoga MA Kortman GAM Boekhorst J Schneeberger S Karanja S. Maternal human milk oligosaccharide profile modulates the impact of an intervention with iron and galacto-oligosaccharides in kenyan infants. Nutrients. (2019) 11:2596. 10.3390/nu11112596 31671757 PMC6893608

[62] Erney RM Malone WT Skelding MB Marcon AA Kleman-Leyer KM O'Ryan ML. Variability of human milk neutral oligosaccharides in a diverse population. J Pediatr Gastroenterol Nutr. (2000) 30:181–92. 10.1097/00005176-200002000-00016 10697138

[63] McGuire MK Meehan CL McGuire MA Williams JE Foster J Sellen DW. What's normal? Oligosaccharide concentrations and profiles in milk produced by healthy women vary geographically. Am J Clin Nutr. (2017) 105:1086–0. 10.3945/ajcn.116.139980 28356278 PMC5402033

[64] Isganaitis E Venditti S Matthews TJ Lerin C Demerath EW Fields DA. Maternal obesity and the human milk metabolome: associations with infant body composition and postnatal weight gain. Am J Clin Nutr. (2019) 110:111–20. 10.1093/ajcn/nqy334 30968129 PMC6599743

[65] Austin S De Castro CA Sprenger N Binia A Affolter M Garcia-Rodenas CL. Human milk oligosaccharides in the milk of mothers delivering term versus preterm infants. Nutrients. (2019) 11:1282. 10.3390/nu11061282 31195757 PMC6627155

[66] Jantscher-Krenn E Treichler C Brandl W Schönbacher L Köfeler H van Poppel MNM. The association of human milk oligosaccharides with glucose metabolism in overweight and obese pregnant women. Am J Clin Nutr. (2019) 110:1335–43. 10.1093/ajcn/nqz202 31504099

[67] Zhou J Wang Y Fan Q Liu Y Liu H Yan J. High levels of fucosylation and sialylation of milk N-glycans from mothers with gestational diabetes mellitus alter the offspring gut microbiome and immune balance in mice. FASEB J. (2020) 34:3715–31. 10.1096/fj.201901674R 31944389

[68] Aksan A Erdal I Yalcin SS Stein J Samur G. Osteopontin levels in human milk are related to maternal nutrition and infant health and growth. Nutrients. (2021) 13:2670. 10.3390/nu13082670 34444830 PMC8402120

[69] Lagström H Rautava S Ollila H Kaljonen A Turta O Mäkelä J. Associations between human milk oligosaccharides and growth in infancy and early childhood. Am J Clin Nutr. (2020) 111:769–78. 10.1093/ajcn/nqaa010 32068776 PMC7138667

[70] Ayoub Moubareck C Lootah M Tahlak M Venema K. Profiles of human milk oligosaccharides and their relations to the milk microbiota of breastfeeding mothers in Dubai. Nutrients. (2020) 12:1727. 10.3390/nu12061727 32526930 PMC7353065

[71] Zhang W Wang T Chen X Pang X Zhang S Obaroakpo JU. Absolute quantification of twelve oligosaccharides in human milk using a targeted mass spectrometry-based approach. Carbohydr Polym. (2019) 219:328–33. 10.1016/j.carbpol.2019.04.092 31151532

[72] Bao Y Zhu L Newburg DS. Simultaneous quantification of sialyloligosaccharides from human milk by capillary electrophoresis. Anal Biochem. (2007) 370:206–14. 10.1016/j.ab.2007.07.004 17761135 PMC2441650

[73] Spevacek AR Smilowitz JT Chin EL Underwood MA German JB Slupsky CM. Infant maturity at birth reveals minor differences in the maternal milk metabolome in the first month of lactation. J Nutr. (2015) 145:1698–708. 10.3945/jn.115.210252 26041675 PMC4516766

[74] Nijman RM Liu Y Bunyatratchata A Smilowitz JT Stahl B Barile D. Characterization and quantification of oligosaccharides in human milk and infant formula. J Agric Food Chem. (2018) 66:6851–9. 10.1021/acs.jafc.8b01515 29799744

[75] Yan J Ding J Jin G Yu D Yu L Long Z. Profiling of sialylated oligosaccharides in mammalian milk using online solid phase extraction-hydrophilic interaction chromatography coupled with negative-ion electrospray mass spectrometry. Anal Chem. (2018) 90:3174–82. 10.1021/acs.analchem.7b04468 29385801

[76] Li J Jiang M Zhou J Ding J Guo Z Li M. Characterization of rat and mouse acidic milk oligosaccharides based on hydrophilic interaction chromatography coupled with electrospray tandem mass spectrometry. Carbohydr Polym. (2021) 259:117734. 10.1016/j.carbpol.2021.117734 33673995

[77] Ma L MacGibbona AKH Jan Mohamedb HJB Loyb S Rowanc A McJarrowa P. Determination of ganglioside concentrations in breast milk and serum from Malaysian mothers using a high performance liquid chromatography-mass spectrometry-multiple reaction monitoring method. Int Dairy J. (2015) 49:62–71. 10.1016/j.idairyj.2015.05.006

[78] Tan S Chen C Zhao A Wang M Zhao W Zhang J. The dynamic changes of gangliosides in breast milk and the intake of gangliosides in maternal and infant diet in three cities of China. Int J Clin Exp Pathol. (2020) 13:2870–88. 33284868 PMC7716121

[79] Yang B Chuang H Yang KD. Sialylated glycans as receptor and inhibitor of enterovirus 71 infection to DLD-1 intestinal cells. Virol J. (2009) 6:141. 10.1186/1743-422X-6-141 19751532 PMC2751754

[80] Nilsson EC Jamshidi F Johansson SM Oberste MS Arnberg N. Sialic acid is a cellular receptor for coxsackievirus A24 variant, an emerging virus with pandemic potential. J Virol. (2008) 82:3061–8. 10.1128/JVI.02470-07 18184708 PMC2259016

[81] Schnabl KL Field C Clandinin MT. Ganglioside composition of differentiated Caco-2 cells resembles human colostrum and neonatal rat intestine. Br J Nutr. (2009) 101:694–700. 10.1017/S0007114508048289 18713482

[82] Le Doare K Holder B Bassett A Pannaraj PS. Mother's milk: a purposeful contribution to the development of the infant microbiota and immunity. Front Immunol. (2018) 9:361. 10.3389/fimmu.2018.00361 29599768 PMC5863526

[83] Moore RE Xu LL Townsend SD. Prospecting human milk oligosaccharides as a defense against viral infections. ACS Infect Dis. (2021) 7:254–63. 10.1021/acsinfecdis.0c00807 33470804 PMC7890562

[84] Pandey RP Kim DH Woo J Song J Jang SH Kim JB. Broad-spectrum neutralization of avian influenza viruses by sialylated human milk oligosaccharides: in vivo assessment of 3'-sialyllactose against H9N2 in chickens. Sci Rep. (2018) 8:2563. 10.1038/s41598-018-20955-4 29416087 PMC5803236

[85] Zevgiti S Zabala JG Darji A Dietrich U Panou-Pomonis E Sakarellos-Daitsiotis M. Sialic acid and sialyl-lactose glyco-conjugates: design, synthesis and binding assays to lectins and swine influenza H1N1 virus. J Pept Sci. (2012) 18:52–8. 10.1002/psc.1415 22052803

[86] Hester SN Chen X Li M Monaco MH Comstock SS Kuhlenschmidt TB. Human milk oligosaccharides inhibit rotavirus infectivity in vitro and in acutely infected piglets. Br J Nutr. (2013) 110:1233–42. 10.1017/S0007114513000391 23442265

[87] Laucirica DR Triantis V Schoemaker R Estes MK Ramani S. Milk oligosaccharides inhibit human rotavirus infectivity in MA104 cells. J Nutr. (2017) 147:1709–14. 10.3945/jn.116.246090 28637685 PMC5572490

[88] Idota T Kawakami H Murakami Y Sugawara M. Inhibition of cholera toxin by human milk fractions and sialyllactose. Biosci Biotechnol Biochem. (1995) 59:417–9. 10.1271/bbb.59.417 7766178

[89] Duska-McEwen G Senft AP Ruetschilling TL Barrett EG Buck RH. Human milk oligosaccharides enhance innate immunity to respiratory syncytial virus and influenza *in vitro*. Food Sci Nutr. (2014) 5:1387–98. 10.4236/fns.2014.514151

[90] Ray C Kerketta JA Rao S Patel S Dutt S Arora K. Human milk oligosaccharides: the journey ahead. Int J Pediatr. (2019) 2019:2390240. 10.1155/2019/2390240 31467568 PMC6699292

[91] Angeloni S Ridet JL Kusy N Gao H Crevoisier F Guinchard S. Glycoprofiling with micro-arrays of glycoconjugates and lectins. Glycobiology. (2005) 15:31–41. 10.1093/glycob/cwh143 15342550

[92] Asadpoor M Peeters C Henricks PAJ Varasteh S Pieters RJ Folkerts G. Anti-pathogenic functions of non-digestible oligosaccharides *in vitro*. Nutrients. (2020) 12:1789. 10.3390/nu12061789 32560186 PMC7353314

[93] Lin AE Autran CA Szyszka A Escajadillo T Huang M Godula K. Human milk oligosaccharides inhibit growth of group B Streptococcus. J Biol Chem. (2017) 292:11243–9. 10.1074/jbc.M117.789974 28416607 PMC5500792

[94] Craft KM Thomas HC Townsend SD. Sialylated variants of lacto-N-tetraose exhibit antimicrobial activity against Group B Streptococcus. Org Biomol Chem. (2019) 17:1893–900. 10.1039/C8OB02080A 30229793

[95] Buescher ES. Anti-inflammatory characteristics of human milk: how, where, why. Adv Exp Med Biol. (2001) 501:207–22. 10.1007/978-1-4615-1371-1_27 11787684

[96] Pacheco AR Barile D Underwood MA Mills DA. The impact of the milk glycobiome on the neonate gut microbiota. Annu Rev Anim Biosci. (2015) 3:419–45. 10.1146/annurev-animal-022114-111112 25387230 PMC4349412

[97] Palmeira P Carneiro-Sampaio M. Immunology of breast milk. Rev Assoc Med Bras 1992. (2016) 62:584–93. 10.1590/1806-9282.62.06.584 27849237

[98] Yolken RH Peterson JA Vonderfecht SL Fouts ET Midthun K Newburg DS. Human milk mucin inhibits rotavirus replication and prevents experimental gastroenteritis. J Clin Invest. (1992) 90:1984–91. 10.1172/JCI116078 1331178 PMC443262

[99] Wang B Brand-Miller J McVeagh P Petocz P. Concentration and distribution of sialic acid in human milk and infant formulas. Am J Clin Nutr. (2001) 74:510–5. 10.1093/ajcn/74.4.510 11566650

[100] Córdova-Dávalos LE Jiménez M Salinas E. Glycomacropeptide bioactivity and health: a review highlighting action mechanisms and signaling pathways. Nutrients. (2019) 11:598. 10.3390/nu11030598 30870995 PMC6471465

[101] Arnold JN Wormald MR Sim RB Rudd PM Dwek RA. The impact of glycosylation on the biological function and structure of human immunoglobulins. Annu Rev Immunol. (2007) 25:21–50. 10.1146/annurev.immunol.25.022106.141702 17029568

[102] Schroten H Stapper C Plogmann R Köhler H Hacker J Hanisch FG. Fab-independent antiadhesion effects of secretory immunoglobulin a on S-fimbriated escherichia coli are mediated by sialyloligosaccharides. Infect Immun. (1998) 66:3971–3. 10.1128/IAI.66.8.3971-3973.1998 9673289 PMC108467

[103] Hejdysz M Kaczmarek SA Rogiewicz A Rutkowski A. Influence of graded dietary levels of meals from three lupin species on the excreta dry matter, intestinal viscosity, excretion of total and free sialic acids, and intestinal morphology of broiler chickens. Ani Feed Sci Technol. (2018). 10.1016/j.anifeedsci.2018.01.015

[104] Owen CD Tailford LE Monaco S Šuligoj T Vaux L Lallement R. Unravelling the specificity and mechanism of sialic acid recognition by the gut symbiont ruminococcus gnavus. Nat Commun. (2017) 8:2196. 10.1038/s41467-017-02109-8 29259165 PMC5736709

[105] Kavanaugh DW O'Callaghan J Buttó LF Slattery H Lane J Clyne M. Exposure of bifidobacterium longum subsp. Infantis to milk oligosaccharides increases adhesion to epithelial cells and induces a substantial transcriptional response. PLoS ONE. (2013) 8:e67224. 10.1371/journal.pone.0067224 23805302 PMC3689703

[106] Meli F Puccio G Cajozzo C Ricottone GL Pecquet S Sprenger N. Growth and safety evaluation of infant formulae containing oligosaccharides derived from bovine milk: a randomized, double-blind, noninferiority trial. BMC Pediatr. (2014) 14:306. 10.1186/s12887-014-0306-3 25527244 PMC4297447

[107] Tarr AJ Galley JD Fisher SE Chichlowski M Berg BM Bailey MT. The prebiotics 3'Sialyllactose and 6'Sialyllactose diminish stressor-induced anxiety-like behavior and colonic microbiota alterations: evidence for effects on the gut-brain axis. Brain Behav Immun. (2015) 50:166–77. 10.1016/j.bbi.2015.06.025 26144888 PMC4631662

[108] Kiyohara M Tanigawa K Chaiwangsri T Katayama T Ashida H Yamamoto K. An exo-alpha-sialidase from bifidobacteria involved in the degradation of sialyloligosaccharides in human milk and intestinal glycoconjugates. Glycobiology. (2011) 21:437–47. 10.1093/glycob/cwq175 21036948

[109] Sela DA Li Y Lerno L Wu S Marcobal AM German JB. An infant-associated bacterial commensal utilizes breast milk sialyloligosaccharides. J Biol Chem. (2011) 286:11909–18. 10.1074/jbc.M110.193359 21288901 PMC3069393

[110] Garrido D Nwosu C Ruiz-Moyano S Aldredge D German JB Lebrilla CB. Endo-β-N-acetylglucosaminidases from infant gut-associated bifidobacteria release complex N-glycans from human milk glycoproteins. Mol Cell Proteomics. (2012) 11:775–85. 10.1074/mcp.M112.018119 22745059 PMC3434770

[111] Vega-Bautista A de la Garza M Carrero JC Campos-Rodríguez R Godínez-Victoria M Drago-Serrano ME. The impact of lactoferrin on the growth of intestinal inhabitant bacteria. Int J Mol Sci. (2019) 20:4707. 10.3390/ijms20194707 31547574 PMC6801499

[112] ELFIN trial investigators group. Enteral lactoferrin supplementation for very preterm infants: a randomised placebo-controlled trial. Lancet. (2019) 393:423–33. 10.1016/S0140-6736(18)32221-9 30635141 PMC6356450

[113] Mastromarino P Capobianco D Campagna G Laforgia N Drimaco P Dileone A. Correlation between lactoferrin and beneficial microbiota in breast milk and infant's feces. Biometals. (2014) 27:1077–86. 10.1007/s10534-014-9762-3 24970346

[114] Griffiths J Jenkins P Vargova M Bowler U Juszczak E King A. Enteral lactoferrin to prevent infection for very preterm infants: the ELFIN RCT. Health Technol Assess. (2018) 22:1–60. 10.3310/hta22740 30574860 PMC6322062

[115] Yoo BB Mazmanian SK. The enteric network: interactions between the immune and nervous systems of the gut. Immunity. (2017) 46:910–26. 10.1016/j.immuni.2017.05.011 28636959 PMC5551410

[116] Moon JS Joo W Ling L Choi HS Han NS. In vitro digestion and fermentation of sialyllactoses by infant gut microflora. J Funct Foods. (2016) 21:497–506. 10.1016/j.jff.2015.12.002

[117] Iraporda C Errea A Romanin DE Cayet D Pereyra E Pignataro O. Lactate and short chain fatty acids produced by microbial fermentation downregulate proinflammatory responses in intestinal epithelial cells and myeloid cells. Immunobiology. (2015) 220:1161–9. 10.1016/j.imbio.2015.06.004 26101138

[118] Donovan SM. Human milk oligosaccharides - the plot thickens. Br J Nutr. (2009) 101:1267–9. 10.1017/S0007114508091241 19079836

[119] Kawashima N Yoon SJ Itoh K Nakayama K. Tyrosine kinase activity of epidermal growth factor receptor is regulated by GM3 binding through carbohydrate to carbohydrate interactions. J Biol Chem. (2009) 284:6147–55. 10.1074/jbc.M808171200 19124464

[120] Wang J Lei B Yan J Li J Zhou X Ren F. Donkey milk oligosaccharides influence the growth-related characteristics of intestinal cells and induce G2/M growth arrest via the p38 pathway in HT-29 cells. Food Funct. (2019) 10:4823–33. 10.1039/C8FO02584C 31318010

[121] Yang C Zhang P Fang W Chen Y Zhang N Qiao Z. Molecular mechanisms underlying how sialyllactose intervention promotes intestinal maturity by upregulating GDNF through a CREB-dependent pathway in neonatal piglets. Mol Neurobiol. (2019) 56:7994–8007. 10.1007/s12035-019-1628-9 31161424

[122] Natividad JM Rytz A Keddani S Bergonzelli G Garcia-Rodenas CL. Blends of human milk oligosaccharides confer intestinal epithelial barrier protection *in Vitro*. Nutrients. (2020) 12:3047. 10.3390/nu12103047 33027993 PMC7599875

[123] Holscher HD Bode L Tappenden KA. Human milk oligosaccharides influence intestinal epithelial cell maturation *In Vitro*. J Pediatr Gastroenterol Nutr. (2017) 64:296–301. 10.1097/MPG.0000000000001274 28114245

[124] Foata F Sprenger N Rochat F Damak S. Activation of the G-protein coupled receptor GPR35 by human milk oligosaccharides through different pathways. Sci Rep. (2020) 10:16117. 10.1038/s41598-020-73008-0 32999316 PMC7528069

[125] Tsukahara T Hamouda N Utsumi D Matsumoto K Amagase K Kato S. G protein-coupled receptor 35 contributes to mucosal repair in mice via migration of colonic epithelial cells. Pharmacol Res. (2017) 123:27–39. 10.1016/j.phrs.2017.06.009 28648739

[126] Farooq SM Hou Y Li H O'Meara M Wang Y Li C. Disruption of GPR35 exacerbates dextran sulfate sodium-induced colitis in mice. Dig Dis Sci. (2018) 63:2910–22. 10.1007/s10620-018-5216-z 30043283 PMC6373462

[127] Schneditz G Elias JE Pagano E Zaeem Cader M Saveljeva S Long K. GPR35 promotes glycolysis, proliferation, and oncogenic signaling by engaging with the sodium potassium pump. Sci Signal. (2019) 12:eaau9048. 10.1126/scisignal.aau9048 30600262 PMC6364804

[128] Pammi M Suresh G. Enteral lactoferrin supplementation for prevention of sepsis and necrotizing enterocolitis in preterm infants. Cochrane Database Syst Rev. (2017) 6:CD007137. 10.1002/14651858.CD007137.pub5 28658720 PMC6481465

[129] Donovan SM Monaco MH Drnevich J Kvistgaard AS Hernell O Lönnerdal B. Bovine osteopontin modifies the intestinal transcriptome of formula-fed infant rhesus monkeys to be more similar to those that were breastfed. J Nutr. (2014) 144:1910–9. 10.3945/jn.114.197558 25320184

[130] Bode L Kunz C Muhly-Reinholz M Mayer K Seeger W Rudloff S. Inhibition of monocyte, lymphocyte, and neutrophil adhesion to endothelial cells by human milk oligosaccharides. Thromb Haemost. (2004) 92:1402–10. 10.1160/TH04-01-0055 15583750

[131] Eiwegger T Stahl B Haidl P Schmitt J Boehm G Dehlink E. Prebiotic oligosaccharides: in vitro evidence for gastrointestinal epithelial transfer and immunomodulatory properties. Pediatr Allergy Immunol. (2010) 21:1179–88. 10.1111/j.1399-3038.2010.01062.x 20444147

[132] Fuhrer A Sprenger N Kurakevich E Borsig L Chassard C Hennet T. Milk sialyllactose influences colitis in mice through selective intestinal bacterial colonization. J Exp Med. (2010) 207:2843–54. 10.1084/jem.20101098 21098096 PMC3005226

[133] Kurakevich E Hennet T Hausmann M Rogler G Borsig L. Milk oligosaccharide sialyl(α2,3) lactose activates intestinal CD11c+ cells through TLR4. Proc Natl Acad Sci USA. (2013) 110:17444–9. 10.1073/pnas.1306322110 24101501 PMC3808656

[134] De Fazio L Beghetti I Bertuccio SN Marsico C Martini S Masetti R. Necrotizing enterocolitis: overview on *in vitro* models. Int J Mol Sci. (2021) 22:6761. 10.3390/ijms22136761 34201786 PMC8268427

[135] Sodhi CP Wipf P Yamaguchi Y Fulton WB Kovler M Niño DF. The human milk oligosaccharides 2'-fucosyllactose and 6'-sialyllactose protect against the development of necrotizing enterocolitis by inhibiting toll-like receptor 4 signaling. Pediatr Res. (2021) 89:91–101. 10.1038/s41390-020-0852-3 32221473 PMC7529714

[136] Jantscher-Krenn E Zherebtsov M Nissan C Goth K Guner YS Naidu N. The human milk oligosaccharide disialyllacto-N-tetraose prevents necrotising enterocolitis in neonatal rats. Gut. (2012) 61:1417–25. 10.1136/gutjnl-2011-301404 22138535 PMC3909680

[137] Masi AC Embleton ND Lamb CA Young G Granger CL Najera J. Human milk oligosaccharide DSLNT and gut microbiome in preterm infants predicts necrotising enterocolitis. Gut. (2020) 16: 10.1136/gutjnl-2020-322771 33328245 PMC9231288

[138] Akin IM Atasay B Dogu F Okulu E Arsan S Karatas HD. Oral lactoferrin to prevent nosocomial sepsis and necrotizing enterocolitis of premature neonates and effect on T-regulatory cells. Am J Perinatol. (2014) 31:1111–20. 10.1055/s-0034-1371704 24839144

[139] Drago-Serrano ME Campos-Rodríguez R Carrero JC de la Garza M. Lactoferrin: balancing ups and downs of inflammation due to microbial infections. Int J Mol Sci. (2017) 18:501. 10.3390/ijms18030501 28257033 PMC5372517

[140] Asztalos EV Barrington K Lodha A Tarnow-Mordi W Martin A. Lactoferrin infant feeding trial_Canada (LIFT_Canada): protocol for a randomized trial of adding lactoferrin to feeds of very-low-birth-weight preterm infants. BMC Pediatr. (2020) 20:40. 10.1186/s12887-020-1938-0 31996186 PMC6988327

[141] Jiang R Lönnerdal B. Evaluation of bioactivities of bovine milk osteopontin using a knockout mouse model. J Pediatr Gastroenterol Nutr. (2020) 71:125–31. 10.1097/MPG.0000000000002702 32141995

[142] Lönnerdal B Kvistgaard AS Peerson JM Donovan SM Peng YM. Growth, nutrition, and cytokine response of breast-fed infants and infants fed formula with added bovine osteopontin. J Pediatr Gastroenterol Nutr. (2016) 62:650–7. 10.1097/MPG.0000000000001005 26465791

[143] Ek-Rylander B Andersson G. Osteoclast migration on phosphorylated osteopontin is regulated by endogenous tartrate-resistant acid phosphatase. Exp Cell Res. (2010) 316:443–51. 10.1016/j.yexcr.2009.10.019 19854170

[144] Møller HK Thymann T Fink LN Frokiaer H Kvistgaard AS Sangild PT. Bovine colostrum is superior to enriched formulas in stimulating intestinal function and necrotising enterocolitis resistance in preterm pigs. Br J Nutr. (2011) 105:44–53. 10.1017/S0007114510003168 20723273

[145] Vázquez E Gil A Rueda R. Dietary gangliosides positively modulate the percentages of Th1 and Th2 lymphocyte subsets in small intestine of mice at weaning. Biofactors. (2001) 15:1–9. 10.1002/biof.5520150101 11673640

[146] Greenspon J Li R Xiao L Rao JN Sun R Strauch ED. Sphingosine-1-phosphate regulates the expression of adherens junction protein E-cadherin and enhances intestinal epithelial cell barrier function. Dig Dis Sci. (2011) 56:1342–53. 10.1007/s10620-010-1421-0 20936358 PMC4140085

[147] Brønnum H Seested T Hellgren LI Brix S Frøkiaer H. Milk-derived GM(3) and GD(3) differentially inhibit dendritic cell maturation and effector functionalities. Scand J Immunol. (2005) 61:551–7. 10.1111/j.1365-3083.2005.01566.x 15963050

[148] Charbonneau MR O'Donnell D Blanton LV Totten SM Davis JC Barratt MJ. Sialylated milk oligosaccharides promote microbiota-dependent growth in models of infant undernutrition. Cell. (2016) 164:859–71. 10.1016/j.cell.2016.01.024 26898329 PMC4793393

[149] Jeon J Kang LJ Lee KM Cho C Song EK Kim W. 3'-Sialyllactose protects against osteoarthritic development by facilitating cartilage homeostasis. J Cell Mol Med. (2018) 22:57–66. 10.1111/jcmm.13292 28782172 PMC5742729

[150] Kang LJ Kwon ES Lee KM Cho C Lee JI Ryu YB. 3'-Sialyllactose as an inhibitor of p65 phosphorylation ameliorates the progression of experimental rheumatoid arthritis. Br J Pharmacol. (2018) 175:4295–309. 10.1111/bph.14486 30152858 PMC6240131

[151] Donovan SM. Human milk proteins: composition and physiological significance. Nestle Nutr Inst Workshop Ser. (2019) 90:93–101. 10.1159/000490298 30865978

[152] Gridneva Z Lai CT Rea A Tie WJ Ward LC Murray K. Human milk immunomodulatory proteins are related to development of infant body composition during the first year of lactation. Pediatr Res. (2021) 89:911–21. 10.1038/s41390-020-0961-z 32438370

[153] Shama S Unger S Pouliot Y Doyen A Suwal S Pencharz P. A human milk-based protein concentrate developed for preterm infants retains bioactive proteins and supports growth of weanling rats. J Nutr. (2021) 151:840–7. 10.1093/jn/nxaa383 33693847 PMC8030702

[154] Wu SL Ding D Fang AP Chen PY Chen S Jing LP. Growth, gastrointestinal tolerance and stool characteristics of healthy term infants fed an infant formula containing hydrolyzed whey protein (63%) and intact casein (37%): a randomized clinical trial. Nutrients. (2017) 9:1254. 10.3390/nu9111254 29144393 PMC5707726

[155] Gridneva Z Tie WJ Rea A Lai CT Ward LC Murray K. Human milk casein and whey protein and infant body composition over the first 12 months of lactation. Nutrients. (2018) 10:1332. 10.3390/nu10091332 30235880 PMC6164442

[156] Tomarelli RM Linden E Durbin GT Bernhart FW. The effect of mucin on the growth of rats fed simulated human milk. J Nutr. (1953) 51:251–9. 10.1093/jn/51.2.251 13097241

[157] Jiang R Liu L Du X Lönnerdal B. Evaluation of bioactivities of the bovine milk lactoferrin-osteopontin complex in infant formulas. J Agric Food Chem. (2020) 68:6104–11. 10.1021/acs.jafc.9b07988 32362125

[158] Andersson EL Hernell O Bläckberg L Fält H Lindquist S. BSSL and PLRP2: key enzymes for lipid digestion in the newborn examined using the Caco-2 cell line. J Lipid Res. (2011) 52:1949–56. 10.1194/jlr.M015685 21865348 PMC3196226

[159] Li X Lindquist S Lowe M Noppa L Hernell O. Bile salt-stimulated lipase and pancreatic lipase-related protein 2 are the dominating lipases in neonatal fat digestion in mice and rats. Pediatr Res. (2007) 62:537–41. 10.1203/PDR.0b013e3181559e75 17805199 PMC3488855

[160] Wang B Brand-Miller J. The role and potential of sialic acid in human nutrition. Eur J Clin Nutr. (2003) 57:1351–69. 10.1038/sj.ejcn.1601704 14576748

[161] Wang HX Chen Y Haque Z de Veer M Egan G Wang B. Sialylated milk oligosaccharides alter neurotransmitters and brain metabolites in piglets: an *In vivo* magnetic resonance spectroscopic (MRS) study. Nutr Neurosci. (2019) 20:1–11. 10.1080/1028415X.2019.1691856 31746283

[162] Waworuntu RV Hanania T Boikess SR Rex CS Berg BM. Early life diet containing prebiotics and bioactive whey protein fractions increased dendritic spine density of rat hippocampal neurons. Int J Dev Neurosci. (2016) 55:28–33. 10.1016/j.ijdevneu.2016.09.001 27603970

[163] Li F Wu SS Berseth CL Harris CL Richards JD Wampler JL. Improved neurodevelopmental outcomes associated with bovine milk fat globule membrane and lactoferrin in infant formula: a randomized, controlled trial. J Pediatr. (2019) 215:24–31.e8. 10.1016/j.jpeds.2019.08.030 31668885

[164] Oh NS Joung JY Lee JY Song JG Oh S Kim Y. Glycated milk protein fermented with lactobacillus rhamnosus ameliorates the cognitive health of mice under mild-stress condition. Gut Microbes. (2020) 11:1643–61. 10.1080/19490976.2020.1756690 32573326 PMC7524334

[165] Jiang R Tran M Lönnerdal B. Recombinant bovine and human osteopontin generated by chlamydomonas reinhardtii exhibit bioactivities similar to bovine milk osteopontin when assessed in mouse pups fed osteopontin-deficient milk. Mol Nutr Food Res. (2021) 65:e2000644. 10.1002/mnfr.202000644 34050612

[166] Huang S Mo TT Norris T Sun S Zhang T Han TL. The CLIMB (Complex Lipids In Mothers and Babies) study: protocol for a multicentre, three-group, parallel randomised controlled trial to investigate the effect of supplementation of complex lipids in pregnancy, on maternal ganglioside status and subsequent cognitive outcomes in the offspring. BMJ Open. (2017) 7:e016637. 10.1136/bmjopen-2017-016637 29025835 PMC5652542

[167] Liu H Radlowski EC Conrad MS Li Y Dilger RN Johnson RW. Early supplementation of phospholipids and gangliosides affects brain and cognitive development in neonatal piglets. J Nutr. (2014) 144:1903–9. 10.3945/jn.114.199828 25411030 PMC4230208

[168] Gurnida DA Rowan AM Idjradinata P Muchtadi D Sekarwana N. Association of complex lipids containing gangliosides with cognitive development of 6-month-old infants. Early Hum Dev. (2012) 88:595–601. 10.1016/j.earlhumdev.2012.01.003 22289412

